# Volatile and bioactive compounds in opercula from Muricidae molluscs supports their use in ceremonial incense and traditional medicines

**DOI:** 10.1038/s41598-017-17551-3

**Published:** 2017-12-12

**Authors:** Bijayalakshmi Devi Nongmaithem, Peter Mouatt, Joshua Smith, David Rudd, Michael Russell, Caroline Sullivan, Kirsten Benkendorff

**Affiliations:** 1 Marine Ecology Research Centre, School of Environment, Science and Engineering, Southern Cross University, Lismore, NSW 2480 Australia; 2 Southern Cross Plant Science, Southern Cross University, Lismore, NSW-2480 Australia

## Abstract

Muricidae molluscs are the source of a valuable purple dye that was traded as a luxury item in the Mediterranean region and by the late Byzantine was reserved for royalty and priests. Less well known is the use of muricid opercula in sacred incense and traditional medicines, although they are still used as rare ingredients today. This study provides the first chemical assessment of opercula from Muricidae, based on several traditional preparation procedures. Chemical analysis of opercula smoke revealed aromatic phenols, which act as fragrance stabilisers and produce a “medicinal” odour. Analysis of lipid extracts revealed pharmaceutically active compounds, including brominated indoles, choline esters and adenosine, consistent with their traditional medical applications. Depending on the preparation procedures, toxic pyridine was also detected. ICP-MS analysis of muricid opercula shows the presence of essential macro and microelements, as well as metals, some of which exceed the recommended safe levels for human use. Nevertheless, these findings support the Muricidae as an historically important marine resource, providing Biblical dyes, medicines and perfume. The opercula contains biologically active compounds and produces smoke containing volatile scent compounds, consistent with their identification as the most likely source of onycha, a controversial ingredient in sacred incense.

## Introduction

**Figure 1:**
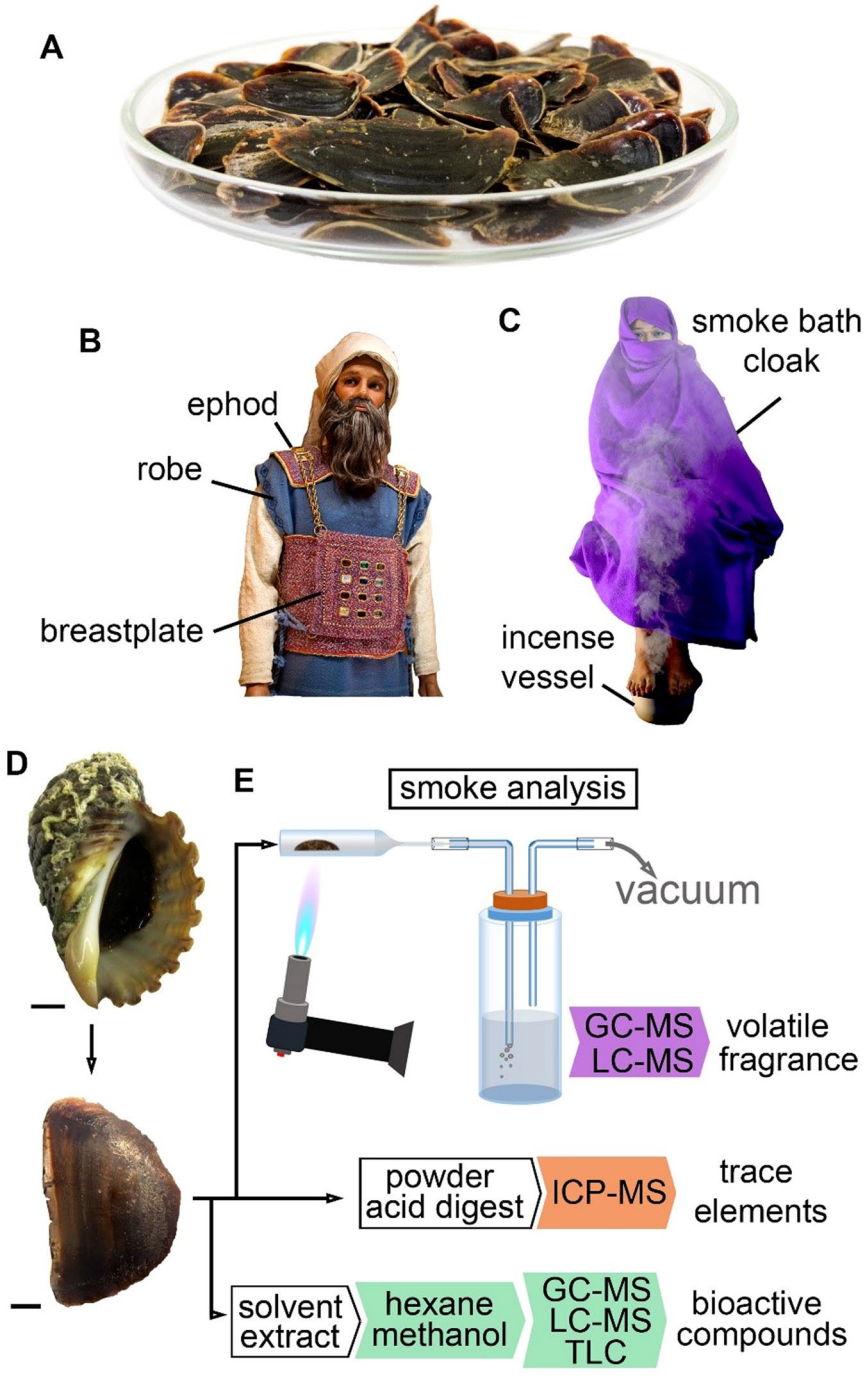
Muricid operculum uses and analysis. (**A**) Muricid opercula (**B**) high priest attire in the Temple of Jerusalem showing robe dyed with Tekhelet blue and the Ephod and breast plate made of woven Shellfish Purple, Tekhelet blue and red dyed yarn. (Modified from Baruch Sterman). (**C**) Demonstration of Sudanese women undertaking a smoke bath. (**D**) *Dicathais orbita* sample (Scale bar = 1 cm). (**E**) Operculum smoke analysis with apparatus used for collecting the volatile compounds from opercula smoke, with the help of a blue butane flame applied to the outer surface of a glass pipette.

Over the last century, there has been much controversy over the identity of onycha, one of the four major ingredients of sacred incense (Exodus 30:34)^1^. The most universally accepted definition for onycha is the operculum (Fig. 1A), a defensive attachment to the foot of gastropod molluscs, which acts to close the shell^2^. However, some controversy surrounds the identification of opercula as the source of an ingredient intended for use in the Holy Tabernacle, on the basis that molluscs, like all shellfish, are included amongst the “unclean” animals of biblical times^3^. Nevertheless, it is well accepted that molluscs of the family Muricidae were the only source of an insoluble purple dye in antiquity, also known as Shellfish purple, Royal purple and Tyrian purple^4^. The use of purple, blue and scarlet twisted yarn in the Tabernacle and garments for the priesthood (Fig. 1B) is specifically mentioned in the Holy Bible (e.g. Exodus 26–28)^1^. More recently, Muricidae molluscs have been confirmed as the source of Tekhelet^5^, the biblical blue traditionally incorporated into priestly garments, including the robe of the Ephod (Fig. 1B), as well as the Jewish tzitzit, tassels of the prayer shawl (Numbers 15:39)^1^. Far from being “unclean”, fabrics adorned with muricid-derived purple and blue threads represented wealth, status and sanctity. Shellfish purple was also used in the textiles of ordinary Roman soldiers and has been found in other Mediterranean archaeological remains, including wall paintings and pottery^6,7^. With this in mind; the perceived “uncleanliness” of molluscs seems to have had little influence on the cultural eagerness to exploit Muricidae products, thus also earning them a conceivable place as a component of sacred incense.

The operculum of neogastropod whelks (predatory sea snails including Muricidae) best fit the description of the “onycha” used in holy incense^3,8^. Onycha is the Greek word for “fingernail” and translated from the Hebrew, “Sheheleth” can be defined as a univalve claw or nail^9^. Other possibilities from plants have been suggested, as more palatable alternatives to the purported “unclean” molluscs, but these are all based on conjecture. These alternatives include benzoin from *Styrax* sp. due to an agreeable vanilla like odour, but which was not locally available; labdanum a resin from *Cistus* sp. because the markings on the flowers are in the shape of a finger nail and it is a product of the Jewish homeland with an agreeable essential oil content^3^; but this is inconsistent with the reported use of onycha as a fixative requiring processing to bring out the odour. Bdellium from *Commiphora* sp. has also been suggested^3^ because it produces a gum that was used in antiquity in incense and produces a sweet spicy smell, although bdellium was commonly identified as a adulterant of myrrh in the spice trade^3,9^. These alternative sources cannot be ruled out, but there is no scientific evidence to support their use in holy incense and none seem to fit the translation of onycha as well as the opercula of neogastropods, which is made of a strong protein similar to the keratin of fingernails. The only other argument against onycha was the supposition that “we know of none bearing any powerful and agreeable odour”^10^. However, also referred to as “*unguis odoratus*” or “sweet hoof”, the opercula of neogastropods have been described as possibly “the most ancient animal derived aromatic to have an extensive global use”^8^.

The ancient muricid dye industry was distributed worldwide, but particularly extensive archaeological evidence stems from the Bronze age or earlier, up until 13^th^ Century AD in the Mediterranean region^7,11–14^. In the city of Tyre, situated in present day Lebanon, ancient dye vats and mounds of broken shells provide evidence for a large scale muricid dye industry^15,16^. The use of operculum in the Mediterranean region possibly evolved as a by-product generated from the purple dye industry, but then became valuable in its own right. The excavation of murex opercula from the cargo of the Bronze Age Uluburun shipwreck (dated to around 1300 BC) provides evidence that Muricidae opercula were traded during antiquity^17^. Thousands of murex opercula were found within amphorae, between copper ingots, recovered from the Uluburun shipwreck and their value has been attributed to their use as ingredients in perfume^17^ and sacred incense^9,18^. The opercula appears to have been traded across the Mediterranean sea during the Greco-Roman period, along with other valuable incense ingredients, including frankincense (olibanum resin from *Bosewellia* trees)^19^.

Burning of operculum is said to produce a strong odour, the scent of which is described as being similar to that of castoreum, the musk derived from the scent gland of mature beavers *Castor* spp., producing an animalic or leathery note^18^. The Arabic term “*azfar al-tibb*” can be translated as sweet hoof^18^, and is used to describe the operculum of *Chicoreus ramosus* (*Murex inflatus*) from Bahrain, which were reported to be the best for fumigation^18,20^. There is historical evidence for the use of opercula across a number of diverse cultural groups separated by time and geographical location (Table 1). Neogastropod opercula are still used today in the incense manufacturing industries of India^8,21^ and as an important ingredient in traditional Sudanese perfumery, particularly as smoke baths for new brides or married women^22^ (Fig. 1C). In contemporary trade, the opercula of the Muricidae species *Chicoreus ramosus* are exported from India to European countries for use in cosmetics, perfumes and medicines^23^.

**Table 1:** Ancient and current traditional medicinal uses of Muricidae opercula.

Country/Ancient Scholar	Source species^#^	Preparation	Pharmaceutical properties (Treatment/curing)	Reference
Ancient Greece/Dioscorides and Galen	*Hexaplex trunculus*, *Bolinus* (*Murex*) *brandaris*, *Stramonita* (*Thais*) *haemastoma*	Crushed and mixed with oil and vinegar	Hearing loss, swollen spleen, depression, menstrual cycle abnormalities and after labour for placenta removal	^24^
Medieval Eastern Mediterranean Genizah	*Chicoreus virgineus* (*Murex anguliferu*)	*Blatta byzantine* Opercula drug	Rheumatism or arthritis, stomach ulcer, skin diseases, teeth problems, eye and ear diseases, tumors, epilepsy, paralysis, purgative and treatment of uterine diseases.	^25^
Kingdom of Bahrein. Ahmad Ibn Muhammad al-Ghafiqi	*Chicoreus ramosus* (*Murex inflatus*)	Fumigation. Smell the smoke produced while placing the operculum on slowly burning charcoal.	Atresia of the uterus	^18,20^
Central Europe and Middle East	*Hexaplex trunculus*	1) Fumigation. 2) Ashes of opercula. 3) Operculum medicine (*Blatta byzantine*) mixed with vinegar.	1) Dislodge the placenta after labour. 2) Wound healing. 3) Reduced swollen spleen	^18,54^
Chinese traditional medicine	*Rapana bezoar*	Decoct* the operculum (10–20 g) and ingest; Ustulate^$^ the shell, grind into powder and apply externally.	Clear heat, expel toxins, remove dampness through diuresis, used to treat strangury (painful & frequent urination), swelling and ulcers on the body surface, hepatic coma, eye diseases and dysentery.	^50,66^
Southern India ancient Sanskrit medical texts	Muricidae opercula	“Nakhi” used in incense and medicinal oil heated in clarified butter or cooked with honey	Destroys poison, destroys certain types of skin diseases, remove phlegm. When used in incense it is said to be like a “prostitute” or “market place charmer”	^8^
Modern India	Muricidae opercula	Opercula oil “Choya Nakh”. Dry distillation of the roasted opercula mixed with a base oil, usually cedarwood oil	Mix a few drops of opercula oil with other incense ingredients. Inhalation of incense smoke to cure stomach pain, liver ailments, epilepsy and irregular menses.	^74,75^

^#^Accepted names are provided according to the World Register of Marine Species^76^ with the alternative names in the original source in brackets, *decoct is to extract by boiling; ^$^ustulate is to blacken or scorch.

Early accounts and treatises on *materia medica* by ancient scholars have highlighted the therapeutic properties of Muricidae operculum (Table 1)^8,18,24–26^. The opercula were used to treat a wide range of illnesses, including swollen spleen, depression, rheumatism or arthritis, stomach ulcers, skin diseases, dental problems, eye diseases, hearing loss, tumours, boils or warts, epilepsy and paralysis, and to expel toxins (Table 1). The opercula were also reported to be useful as purgatives and laxatives^18,20,25^ and were specifically used for the treatment of gynaecological problems, including uterine diseases, menstrual cycle abnormalities and removal of the placenta post-partum (Table 1). Only two previous studies have investigated the bioactivity of muricid opercula and both report mild antimicrobial activity^27^ and muscle relaxing properties^28^. However, a recent review of biologically active compounds isolated from other Muricidae tissues has revealed a broad suite of relevant pharmacological properties associated with the precursors of Shellfish purple^29^. These compounds are known to be stored in the hypobranchial glands of Muricidae^30^ and it is uncertain whether they also occur in the operculum.

To reconcile the long and culturally diverse practice of utilising opercula in traditional medicine and religious ceremony, further research on the bioactive secondary metabolites^31^ and volatile compounds from opercula is warranted. In this study, *Dicathais orbita* (Fig. 1D) was used as a model species of Muricidae because it has been subject to extensive chemical and biological studies^30^. Several methods were used to prepare the operculum (Fig. 1E), based on historical and contemporary methods for processing the operculum for use in the production of incense and traditional medicines (Table 1). Comparative analyses were undertaken on smoke extracts from the opercula of *Chicoreus ramosus* as a representative species historically employed as an incense and perfume ingredient from antiquity to the present day. The volatile and bioactive compounds detected in this study (summarized in Supplementary Table S1) help substantiate the persistent use of Muricidae operculum by a variety of cultural groups, in incense and sacred practices, perfumery and traditional medicines.

## Results and Discussion

### Operculum fumigation: Volatile compounds in the operculum smoke extracts

The use of operculum smoke has been prevalent among a number of cultures throughout antiquity, most notably for religious customs and as a medicinal treatment. Fumigation was used in the Middle East and Central Europe, as well as in historical and contemporary Sudan, particularly for the treatment of gynaecological disorders (Table 1). Operculum smoke is still valued today in Indian and Central European perfumery.

**Figure 2:**
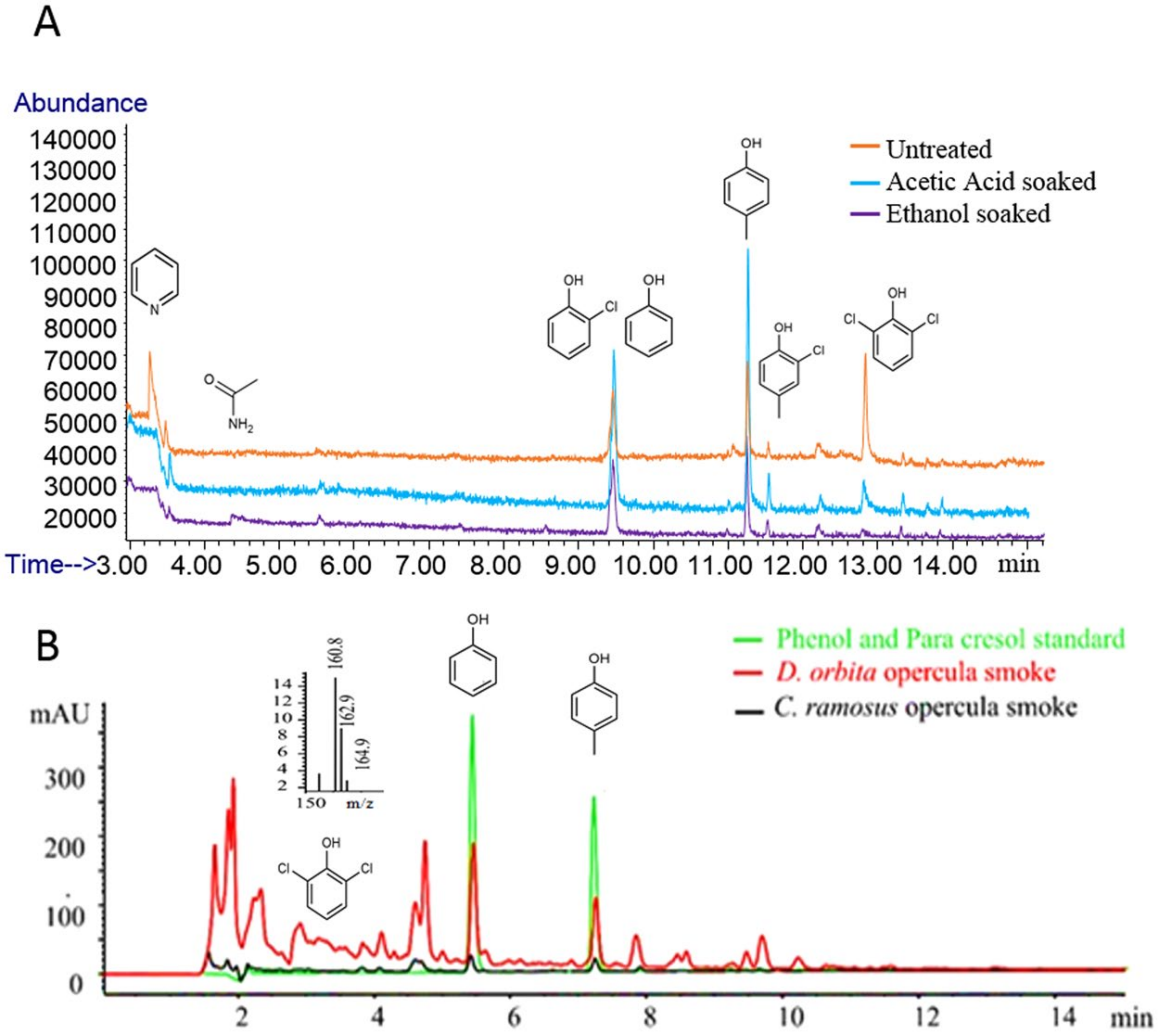
Analysis of the opercula smoke extracts. (**A**) GC-MS of the smoke extracts of *Dicathais orbita* opercula showing total ion current chromatograms overlaid for three different opercula preparations, untreated (orange line), soaked in 5% acetic acid (blue line) or soaked in 70% ethanol (purple line). (**B**) Preliminary LC-MS comparison of opercula smoke extracts from *Chicoreus ramosus* (black line) and *D*. *orbita* (red line), to reference standards for phenol and para-cresol (green line). The HPLC chromatograms are presented from the diode array detected at 210 nm. The X axis represents the retention time (minutes) and Y axis represents the absorbance units (mAU) from the diode array. Phenol and cresol can not be detected in the total ion current (Supplementary Fig. S2A), but the retentions times (phenol r.t 5.48 min and cresol r.t 7.17 min), UV-Vis spectra and selected ion monitoring (Supplementary Figure S2) correspond to synthetic reference standards. A dichlorophenol is tentatively identified in the extracts (r.t. 3.021 min) by the mass spectra (inset, UV-Vis in Supplementary Figure S2 B, C). Due to a very limited number of *C*. *ramosus* opercula, these samples were much more dilute than *D*. *orbita*. Compound structures are representative of possible isomers for the phenols.

The volatile compounds detected in the opercula smoke included a number of common ingredients used for medicinal, fragrance and industrial purposes (Table 2). When trapped in 25% ethanol after burning in a purpose built apparatus (Fig. 1E), gas chromatography mass spectrometry (GC-MS) analysis revealed a predominance of aromatic compounds in the operculum smoke (Fig. 2A, Supplementary Fig. S1). Cresol and phenol, which co-eluted with chlorophenol (Fig. 2A) were consistently present as major constituents (20–40% of smoke extracts), irrespective of operculum pre-treatment (Table 2). A range of more complex phenols occur in castoreum^32^, but simple phenols like cresol could contribute to the musty, leathery note from both of these sources of animalic scents^18^. Comparative analysis of the opercula smoke of *D*. *orbita* and *Chicoreus ramosus* by liquid chromatography mass spectrometry (LC-MS), confirmed the presence of phenol and cresol based on matching retention times (Fig. 2B), and UV-Vis profiles to reference standards (Supplementary Fig. S2). These compounds are not detected in the total ion current using electrospray ionisation, in either the positive or negative mode, however we were able to detect small peaks using selected ion monitoring at *m/z* 94 for phenol and 109 for cresol, in reference standards (Supplementary Fig. S2a) and opercula smoke samples (Supplementary Fig. S2b and c). Both para-cresol and phenol are used as antioxidants in fragrances^33,34^, to help prevent oxidative degradation of essential oils^35^. The presence of these anti-oxidant compounds in the smoke is therefore consistent with the reported role of opercula in traditional fumigation practices from Sudan, where the operculum is said to help stabilise the fragrant ingredients and contribute to the long lasting smell of the perfume^22^.

**Table 2:** Volatile compounds detected by GCMS from the muricid *Dicathais orbita* opercula smoke after different preparation treatments.

Compound	Structure^#^	Retention time (min)	Major ion m/z	Relative abundance (% compound ± SD)^$^			Permissible exposure limit (ppm)	Bioactive properties or uses
				Untreated opercula	70% Ethanol soaked	5% Acetic acid soaked		
Pyridine		3.29	79	39.21 (±3.09)	ND	ND	15^41^	toxic compound^39^, increased oestrous cycle^41^, reduced sperm motility^40^, fungicide and insecticide^77^, gastrointestinal upset and central nervous system (CNS) depression at high level exposure^78^
Acetamide		4.38	59, 44	ND	2.66 (±0.25)	ND	NA	skin and hair cosmetic ingredient^79^
Chloro-phenol^*^		9.4	130, 128, 100	25.14 (±0.38)*	47.08 (±1.28)*	37.07 (±1.09) ^*^		odour quality described as ‘medicinal’^80^
Phenol^*^		9.45	94				5^81^	fragrances and as an antioxidant^33,34^, oral anaesthetic drug^82^, treatment of ingrown toenails^82^
Para cresol		11.2	107, 90	21.08 (±0.83)	35 (±1.58)	42.56 ± 1.04)	5^83^	ingredient in fragrances and an antioxidant^33,34^
Chloro-methylphenol		11.52	144, 142, 107	4.89	5.08 (±0.39)	4.99 (±0.27)	NA	estrogen receptor binding activity^37,84^
Dichloro- phenol		13.3	166, 164, 162	traces	2.93 (±0)	3.36 (±0)	NA	odour quality described as ‘medicinal’^80^

^#^Representative structures only, the specific isomers have not been positively identified for the relevant phenols.

*Although 2-chlorophenol eluted slightly earlier than phenol, due to the relatively large amount of phenol, these compounds co-eluted as overlapping peaks in most of the samples. Therefore, the relative abundance for phenol and 2-chlorophenol have been quantified together by integrating the combined area under the curve. It is estimated that approximately 80% of this combined area is due to phenol based on the peak profiles of the mass ions for *m/z* 94 vs *m/z* 128 for chlorophenol.

^$^The total relative abundance for the compounds presented here does not add up to 100% due to the presence of other unidentified compounds in the extracts. ND – Not detected over the minimum detection level (<0.1%), traces refers compounds that can be confirmed by MS but with a relative abundance of 0.1–0.5% in the GC, NA- Not Available.

Chlorinated phenols, including chloro-phenol, chloro-methyl-phenol and a dichloro-phenol were also detected with GC-MS analysis of the *D*. *orbita* opercula smoke samples (Fig. 2A, Table 2 and Supplementary Fig. S1). LC-MS analysis of the smoke extracts also confirmed the presence of a dichloro-phenol in *D*. *orbita* and *C*. *ramosus* operculum smoke (Fig. 2B) based on the UV profile for the peak at 3.021 min and [M − H]^−^ ions at *m/z* 160.8, 162.9, 164.9 detected in negative ion mode (Supplementary Fig. S2B, C). A previous sensory study on phenols has revealed that these chlorinated phenols can be detected as odours at very low concentrations, with an odour quality described as ‘medicinal’, which would be compatible with use in ceremonial incense employed for spiritual cleansing. The presence of chlorinated phenols is consistent with the high levels of halogenation often found in marine natural products^36^, although only brominated secondary metabolites have been previously reported as occurring in *D*. *orbita* and other muricids^29^. The volatile compound 2-chloro-4-methylphenol is reported to have estrogen receptor binding activity^37^, which may explain the use of opercula smoke for gynecological treatments in Sudan^22^ and ancient Greece^24^. For example, it is a tradition for Sudanese women to undergo *Dukhan* (a smoke bath with incense containing opercula) regularly after marriage^38^ (Fig. 1C). Estrogen-like activity may also explain why in Sanskrit the incense made from *nakha* (opercula) were described as having “charming” or “prostitute-like” properties^8^.

Traditional methods of pre-treating the operculum prior to burning were found to influence the composition of volatile compounds in the resulting smoke (Fig. 2A). In particular, pyridine was only detected in the smoke of untreated opercula, where it was a dominant compound detected (>30%) (Table 2). Pyridine is a toxic compound^39^, thought to affect the mammalian male reproductive system, which may also explain the more frequent traditional uses of operculum smoke and medicines specifically by women. Male mice exposed to drinking-water containing pyridine with minimum doses of 100 ppm showed reduced sperm motility and testicular weight^40^. Conversely, pyridine was found to increase the oestrous cycle length at the highest dose level in female mice^41^. The potential to increase or enhance the period in which females are in oestrous (fertile and sexually receptive) may be one reason why the opercula smoke baths are promoted for use only by married women in the Sudanese culture^22^. Pyridine, however, has also been found to have toxic effects on the liver^39^. Washing the muricid opercula with vinegar (e.g. 5% acetic acid) or alcohol (e.g. 70% ethanol) prior to burning can effectively remove this toxic compound, suggesting that the traditional methods of preparation^22,42^ may be essential for reducing harmful effects, in addition to removing the associated fishy smell.

### Oil-based operculum medicines: Natural products in the lipophilic extracts

Extraction of the metabolites using oil based solvation was a common practice in ancient Greece in 1st Century AD. This operculum oil medicine was reported as being sold in medieval Jewish pharmacies from 11th–14th Century AD predominantly for gynaecological disorders, depression and a range of inflammatory diseases (Table 1)^29^. In some Middle Eastern countries the opercula was ground into a powder then heated in a pan with oil or ghee, to avoid overheating and burning the opercula by direct contact. Both the oil and heated opercula were used as incense ingredients^42^. In ancient Greece the crushed operculum mixed with oil and vinegar was used to treat cases of swollen spleen and menstrual cycle abnormalities^24^.

**Figure 3:**
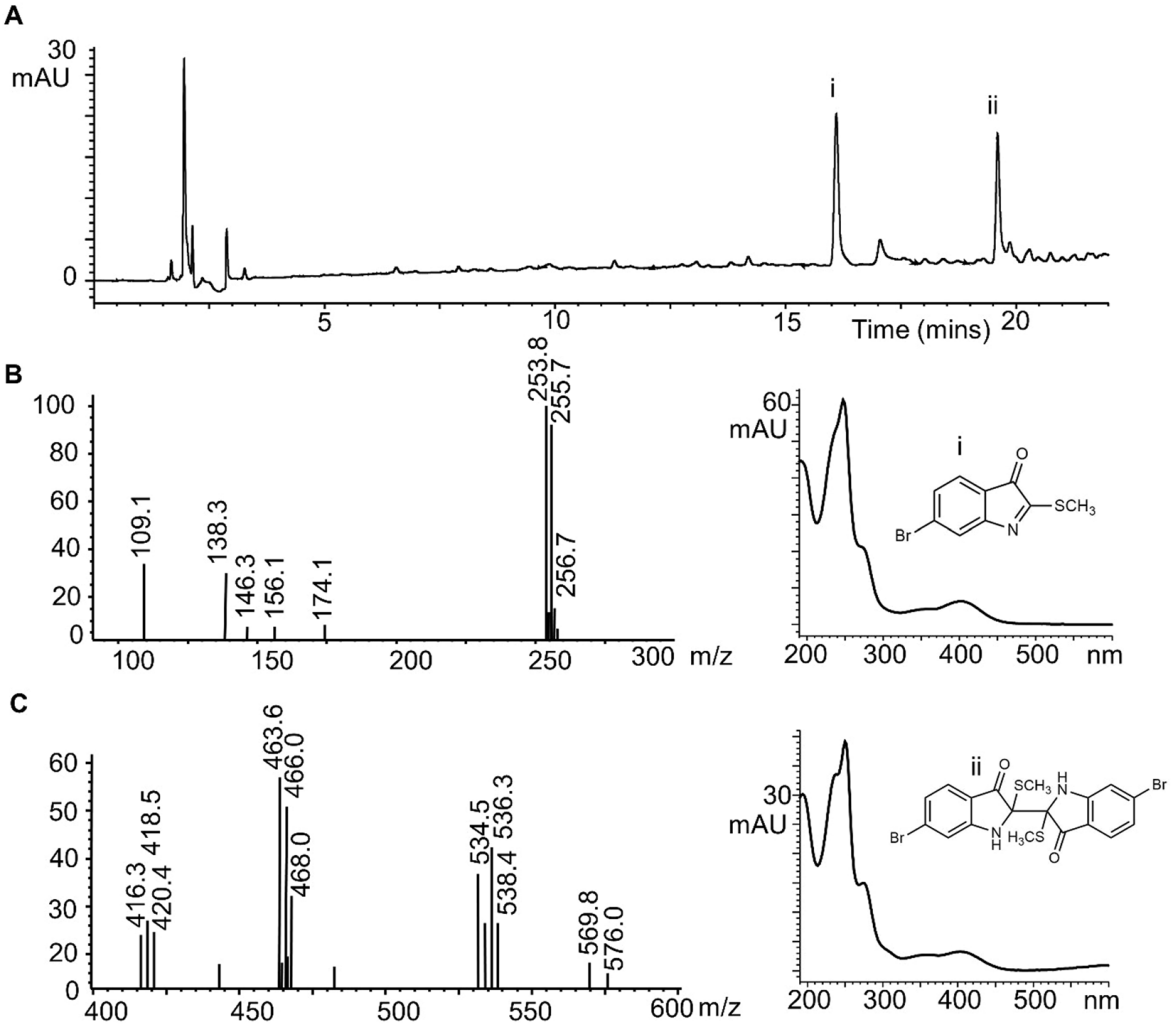
Chemical analysis of *D*. *orbita* opercula hexane extract showing. (**A**) Liquid chromatogram at 280 nm. The X axis represents the retention time (minutes) before the compounds were detected by the diode array. Y axis represents the absorbance units (mAU) from the diode array. The resulting peaks are identified by mass spectrometry and UV-Vis spectra as (**B**) i Tyrindoleninone. (**C**) ii Tyriverdin (Na+ adduct).

To examine the lipophilic compounds likely to be extracted in oil, we used hexane extracts of *D*. *orbita* operculum. Among the lipophillic compounds detected by GC-MS (Supplementary Table S2), the bioactive brominated indole, tyrindoleninone (6-bromo-2methylthio-3H-indol-3-one, Supplementary Fig. S3) was tentatively identified. LC-MS analysis of the hexane extract (Supplementary Fig. S4) further supports the presence of tyrindoleninone (**i**) in the opercula, with a small peak detected at 280 nm after 16.119 min (Fig. 3A) with product ions [M − H]^+^ at *m/z* 254, 256 ^79^Br ^81^Br (Fig. 3B). Indoles can undergo hydrogen abstraction to form [M − H]^+^ ions under ESI-MS when present in complex mixtures^43^. Tyrindoleninone is an intermediate precursor to Shellfish purple, which has been well documented from the hypobranchial glands and egg masses of *D*. *orbita* and other Muricidae^4,30,44–48^. Consistent with the use of opercula for menstrual problems and diseases of the uterus, tyrindoleninone has steroidogenic activity, stimulating estrogen synthesis and modulating progesterone synthesis, as well as specifically inhibiting the proliferation of female reproductive cancer cells^29,49^. The brominated indole dimer tyriverdin (**ii**) was also detected by LC-MS at 19.665 min (Fig. 3A), with molecular ions for the Na^+^ adduct [M + Na]^+^ at *m/z* 534, 536 & 538 and fragment ions at *m/z* 416, 418 & 420 [M-2SCH_3_]^+^ and *m/z* 463, 465 & 467 [M-SCH_3_]^+^ (Fig. 3C). Both tyriverdin and tyrindoleninone exhibit antimicrobial activity^44^, which may contribute to the traditional Chinese uses of opercula for the treatment of dysentery and Middle Eastern uses of opercula in wound healing treatments (Table 1).

### Opercula decoctions and alcoholic extracts: Natural products in the polar operculum extracts

Aqueous and alcoholic solvation is another common process for preparing opercula in traditional medicines. Water or alcohol based decoctions were used in traditional Chinese medicine for treating tumours and ulcers, expelling toxins from the body and relieving inflammatory conditions^50^; whereas vinegar and alcohol mixtures were used in Central European, Middle Eastern and ancient Greek medicine for arthritis, dental diseases, epilepsy and removal of the placenta (Table 1). The specific methods for preparing the opercula medicine sold in Jewish pharmacies during medieval 11–14th centuries^25^ are unknown, but might also have involved extraction in water or alcohol. Therefore, we also examined polar (methanol) extracts of *D*. *orbita* opercula (Fig. 1E).

**Figure 4:**
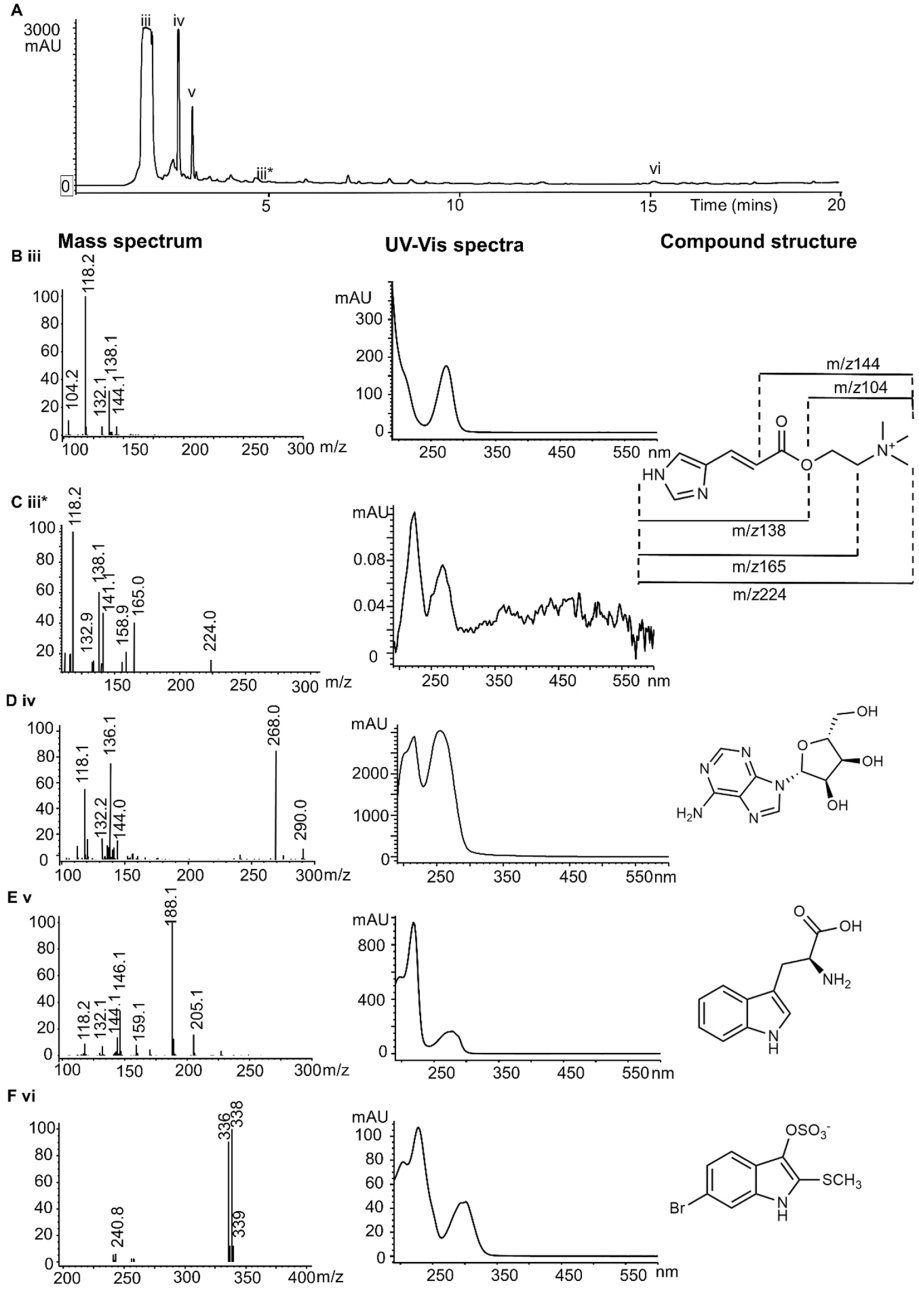
Chemical analysis of *D*. *orbita* opercula methanol extract showing. (**A**) Liquid chromatogram at 280 nm. The X axis represents the retention time (minutes) in the C18 column before the compounds were detected by the diode array. Y axis represents the absorbance units (mAU) from the diode array. The resulting peaks are identified by MS (left) and UV-Vis spectra (right) by comparison to pure reference standards as (**B**) iii choline and urocanic acid fragment ions (**C**) iii* Murexine (**D**) iv Adenosine, (**E**) v Tryptophan, and (**F**) iv Tyrindoxyl sulfate.

Among the polar compounds detected by GC-MS, biologically active compounds of interest include the nucleobases thymine and adenine (Supplementary Table S1 and Fig. S4). Thymine is reported to have antitumor activity against mammary adenocarcinoma (breast cancer), whereas adenine has immunomodulatory activity^51,52^. Using LC-MS (Supplementary Fig. S6), the purine nucleoside adenosine (**iv**) was confirmed as a dominant compound in the methanol extract (Fig. 4A), with retention time 2.84 min (consistent with the reference standard) and adduct ion [M + H]^+^ at *m/z* 268 (C_10_H_14_N_5_O_4_) with fragment ions *m/z* 136 and 144 (Fig. 4D). Adenosine has anti-inflammatory activity and muscle-relaxing properties that are reported to be safe but with similar effects to drugs like verapamil^53^. This is consistent with previous reports on the muscle relaxing activity of muricid opercula extracts from India, which were also suggested to have similar activity to verapamil^28^. These anti-inflammatory, analgesic and muscle relaxing compounds may partly explain traditional medicinal applications in Central Europe and the Middle East for the treatment of arthritis, dental problems, epilepsy and dislodging the placenta after birth^18,24,54^. Phenol, detected in the smoke extracts (Table 2), is also used as an oral anaesthetic drug^55^.

The amino acid tryptophan (**v**) was detected in the methanol extract at 3.321 min (Fig. 4E v) and confirmed by comparison to a synthetic standard, with the adduct ion [M + H]^+^ at *m/z* 205 (C_11_H_13_N_2_O_2_) and major fragment ion at *m/z* 188 (Fig. 4Ev). Tryptophan is the biosynthetic precursor to most indoles, including tyrindoxyl sulfate, the ultimate precursor of Shellfish purple in *D*. *orbita* ^30^. LC-MS analysis confirmed presence of a small peak corresponding to tyrindoxyl sulfate (**vi**) at 15.114 min (Fig. 4Fvi), which matches the purified compound isolated from the hypobranchial glands^56^, with a molecular mass in negative ion mode for [M]^−^ at *m/z* 336, 338 [C_9_H_8_BrNO_3_OS_2_]^−^. Thin layer chromatography (TLC) developed with Dragendorff’s reagent confirmed the presence of tyrindoxyl sulfate (R_f_ = 0.23), which gradually turns purple after exposure to hydrochloric acid (Supplementary Fig. S7)^57^. Tyrindoxyl sulfate (**vi**) can be hydrolysed in acid to form the bioactive brominated indole precursors of Shellfish purple (**vii**)^30^, including tyrindoleninone and 6-bromoisatin, which have been found to prevent the formation of early stage colon cancer in rodent models of disease progression^58^. These intermediate precursors will react to form the dye pigment 6,6′dibromoindirubin, a specific GSK-3 inhibitor^59^ that has led to the development of the commercially available pharmaceutical agent 6-bromoindirubin-3′-oxime (6BIO), a compound with potential practical applications in regenerative medicine^60^. The presence of these brominated indoles may also therefore provide some rationale for the ancient use of operculum derived medicines for the treatment of tumours, stomach ulcers and a range of other medical conditions (Table 1)^25^.

In the hypobranchial glands of *D*. *orbita*, the brominated indole precursors of Shellfish purple are stored as a salt of the choline ester murexine^46^. Murexine exhibits muscle relaxing activities^61^ and along with adenosine has analgesic activity^62^, which could explain the widespread use of muricid opercula in the treatment of women’s problems (Table 1). TLC with Dragendorff’s reagent confirmed a yellow/orange pigmented spot corresponding to murexine (**viii**) (r.f = 0.29, Fig. S7)^61^, as well as a purple pigmented spot corresponding to choline (**ix**) (r.f = 0.32, Fig. S7)^45^. LC-MS analysis of the methanol extracts also shows the presence of a minor peak at 4.860 min (Fig. 4Ciii*) that corresponds to murexine with molecular ion at *m/z* 224 [C_11_H_18_N_3_O_2_]^+^ and fragment ions at *m/z* 165 and 159 (acryloylcholine, [C_8_H_18_NO_2_]^+^ (Fig. 4Ciii*) in positive ion mode. Acryloylcholine fragment ions have been previously reported in association with murexine isolated from muricids^63^. Murexine appears to be a minor constituent in opercula in comparison to the hypobranchial gland of *D*. *orbita* ^56^. However, a major peak at 2.041 min (Fig. 4Biii) also appears to be associated with murexine or a related choline ester, with fragment ions at *m/z* 144, 138 and 104 (Fig. 4Biii). The fragment ion at *m/z* 104, corresponds to choline [C_5_H_14_NO]^+^ and *m/z* 144 appears to be a choline ester fragment (Fig. 4Biii and 4C iii*). The fragment ion for the urocanic acid (4-imidazoleacrylic acid) moiety of murexine (C_6_H_6_N_2_O_2_) is also detected with *m/z* 138 in both the murexine peak and related choline ester (Fig. 4Biii and Ciii*). The choline ester at 2.041 min has the same UV profile and retention time as a reference standard for urocanic acid with [M + H]^+^ at *m/z* 139. This urocanic acid ion has also been reported from mass spectrometry imaging mapping studies and shows a specific distribution in the hypobranchial gland of *D*. *orbita* ^64^. Previous studies have reported that the identification of murexine derivatives is difficult due to the series of fragment ions characteristic of the N-methylimidazolyl moiety^65^. Nevertheless, both the TLC and LC-MS analyses support the presence of muscle-relaxing choline esters in the opercula extracts.

### Opercula powder and ashes: Trace element analysis

The ashes of the ground opercula are used in several traditional medicinal preparations (Table 1), including Chinese traditional medicine^50,66^. Trace element analysis from muricid opercula shows a mix of essential macro and microelements, as well as the presence of heavy metals (Table 3), some of which appear beyond the recommended safe levels for human consumption. This may not have been a problem in pre-industrial times and may depend on local geochemistry, but predatory marine molluscs such as Muricidae can bioaccumulate heavy metals from polluted waters^67^. In *D*. *orbita* opercula, metals such as arsenic, chromium, selenium, aluminium, boron and vanadium exceed 10x the maximum tolerable limit (Table 3). In particular the total arsenic was 25 mg/kg, although our analysis has not distinguished between the toxic inorganic and organic forms of arsenic. Previous studies on shellfish indicate that inorganic arsenic generally constitutes <1% of the total arsenic^68,69^ and if this holds true for opercula it would be below the safe limits for inorganic arsenic, which is recommended as a maximum of 1 mg/kg in mollusc flesh^70^. Furthermore, the actual dose taken in ground operculum medicines is unknown, but is unlikely to be more than a few grams. Nevertheless, some caution is required for any regular direct ingestion of the operculum or ash.

**Table 3:** Trace element composition of the muricid (*Dicathais orbita*) opercula.

Element (mg/Kg)	Opercula Powder (mg/kg)
Calcium	6356
Sulfur	6300
Sodium	3597
Potassium	1614
Magnesium	918
Phosphorus	689
Iron	311.0
Silicon (acid soluble)	289
Aluminium	40.3^#^
Total arsenic^^^	25.7^#^
Selenium	23.3^#^
Boron	16.7^#^
Zinc	14.4
Copper	6.0
Chromium	3.1^#^
Manganese	2.8
Vanadium	2.0^#^
Lead	0.3
Molybdenum	0.3
Barium	0.2
Cadmium	0.2
Silver	0.2
Cobalt	0.1
Mercury	0.002

^#^Element > 10x maximum tolerable limit^70,85^.

^^^1 mg/kg inorganic arsenic permitted in Mollusc.

## Conclusions

Overall, this study highlights the presence of volatile bioactive compounds that may have contributed to the traditional use of Muricidae operculum and its smoke in perfumery and therapeutics. Our results also provide the first evidence for the presence of odorous compounds and fixatives in the operculum smoke that would complement the other fragrant ingredients in holy incense (frankincense, galbanum and “stacte” – most likely thought to be myrrh resin). The tradition of dying with Muricidae Shellfish purple and Tekhelet blue were both lost from history with the fall of the Byzantine court in Constantinople and the destruction of the Hebrew Second Temple in Jerusalem, respectively, only to be “rediscovered” centuries later by careful chemical research^5,11,71^. It seems feasible that simultaneous to the loss of these mollusc dye industries, the identity of onycha, the fourth ingredient in “holy incense”, was also lost. Due to the high value and religious significance of these Muricidae industries, the processes were probably deliberately kept secret. Whilst we cannot retrospectively conclusively identify muricid opercula as the original source of onycha, our chemical analyses do confirm fixative properties and medicinal scent qualities consistent with this type of ceremonial use. Future work on any archaeological samples, such as the excavated incense burners from Palestine^72^, could include investigations for Muricidae chemical markers, such as chlorinated phenols, brominated indoles and choline esters to help confirm opercula as the source of Onycha. It is unlikely that any volatile smoke compounds would persist overtime, but there is a chance that traces of the highly stable Shellfish purple 6,6-dibromoindigo may still be detected.

Our study also establishes the presence of known bioactive compounds in lipophilic and alcoholic extracts of muricid opercula. From this research, we find a strong correlation between the traditional medicinal uses of muricid opercula and the reported biological activity of the compounds identified from muricid opercula smoke and extracts. Further analysis on the effective dose received during the traditionally administered route would shed more light on the potential therapeutic effects. However, some toxic compounds, including metals and pyridine were also detected in the untreated operculum and its smoke, suggesting caution is required when preparing and applying these traditional medicines. Washing the operculum in alcohol is recommended prior to inhalation of the smoke. Furthermore, lipophilic extracts prepared in oil that contain bioactive brominated indoles without the toxic pyridine or metals are likely to be safer than whole ground operculum or ashes for incorporation into medicines. Overall, the opercula of the muricid is a low cost by-product from Muricidae fisheries that appears to have been traditionally valued as an ingredient in incense and medicines. Assuming harvest is environmentally sustainable, Muricidae could again be value-added as ingredients in modern western perfumery and alternative medicines.

## Materials and Methods

### Chemicals

Ethanol, acetic acid, *n-*hexane, *n*-butanol and methanol were HPLC grade (Sigma-Aldrich, St. Louis, USA). Ultrapure water was obtained using Milli-Q (Merck, Kenilworth, USA). Reference standards included phenol (Sigma-Aldrich; 99% purity), para cresol (Sigma-Aldrich; 99% purity), urocanic acid (4-Imidazoleacrylic acid; Sigma; 99% purity), choline bitartrate (Sigma; 99% purity), L-tryptophan (Sigma; with ≥98% purity) and adenosine (Olchemim; 98% purity). Dragondorff’s reagent was used for TLC visualisation (Fluka; St. Louis, USA).

### Sample collection and dissection

*Dicathais orbita* (Gmelin, 1791) adult specimens (Fig. 1D) were collected under the permit number F89/1171–6.0 and P10/0069–1.0 issued by Department of Primary Industries, New South Wales Government, Australia. Reference specimens are lodged in the Molluscan Reference Collection in the National Marine Science Centre, Coffs Harbour, NSW, Australia. The live whelks were collected during low tides throughout 2014 from intertidal rocky reefs along 200 km approximately of the coast of northern NSW from Coffs Harbour to Lennox Head. In total 410 opercula (42 g) were used for this study, which were all collected as a by-product from other studies on *D. orbita* tissues, thus maximizing the use of the whelks and reducing the overall environmental impact of collections from our laboratory.

The hard shell of frozen *D*. *orbita* was removed according to methods described by Westley and Benkendorff^57^. Shells were fractured using a bench vice with pressure applied at the point between the primary body whorl and spire. After removing the shell (Fig. 1D), opercula (Fig. 1A) were carefully separated from the snail flesh using a sharp scalpel and placed inside a fume cabinet to dry for one week.

In addition, opercula were collected from three specimens of *Chicoreus ramosus* (Linneus 1758) obtained from the fishing village Threspuram, Southeast coast of India and identified against reference specimens in the Central Marine Fisheries Institute Museum, Tuticorin, India. These three opercula were dried, combined and crushed into a powder (6 g) for smoke analysis.

### Opercula sample preparation for smoke analysis

Use of the opercula in traditional medicine and perfumery involves certain traditional preparations to derive the scent^42^. These different methods of preparation are all likely to influence the final chemical composition of the extracts and burning properties of the operculum. In Japan and China, opercula are treated with alcohol or vinegar in order to remove the fishy aroma, followed by roasting on a fire, then ground into powder^22^. The opercula of the muricid was observed to produce a fishy odour before treatment. Therefore we trialed soaking the opercula in various concentrations of ethanol (alcohol) and 5% acetic acid (to replicate use of vinegar in some traditional preparations) for different lengths of time, then monitoring until the fishy smell was removed. Four groups of dried opercula weighing 0.27 g (approx.) each were taken and treated with 25 ml ethanol (70%), 25 ml ethanol (50%), 25 ml ethanol (25%) and 25 ml acetic acid (5%) respectively. It was observed that the fishy smell was removed from the opercula treated with ethanol (70%) and acetic acid (5%) after one week. The opercula treated with ethanol (50%) took two weeks to get rid of the fishy smell and ethanol with (25%) took three weeks. Ethanol (70%) and acetic acid (5%) were therefore used for treatment of opercula prior to smoke analysis.

For smoke analysis three groups with 10 g each of dried *D*. *orbita* opercula were used. The first group of opercula (10 g) were soaked in 100 ml ethanol (70%) for one week. The ethanol was decanted and the opercula were rinsed with ethanol (70%) then air dried for a further three days. A second group of opercula (10 g) were soaked in 100 ml acetic acid (5%) for one week and then the acetic acid was decanted and the opercula were then rinsed with acetic acid (5%) and allowed to air dry for three days. The third group of opercula (10 g) was left untreated. In order to remove all the moisture, the opercula from the three groups were stored inside separate desiccators until processing. The dried opercula were pulverized into powder using a ball grinder (Retsch MM301, Haan, Germany) for 30 seconds at a frequency of 30 Hz and stored inside an airtight container for further analysis. Similarly, the dried *C*. *ramosus* opercula were also split into three groups (2 g) for untreated, 70% ethanol soaked and 5% acetic acid soaked preparations, prior to burning.

### Opercula burning and smoke collection

In order to collect the volatile compounds from the smoke of burnt opercula, a special purpose built smoke trap apparatus was constructed (Fig. 1E). Two ‘L-shaped’ sections of steel tubing were mounted protruding through the lid of a 10 ml glass vial (Agilent, Santa Clara, USA). One section of the steel tubing was positioned with the internal end near to the base of the vial (below the intended level of the trapping solvent), with a purpose machined Teflon adapter fitted to the external end to allow connection to the narrow end of a glass Pasteur pipette. The other section was positioned above the level of the trapping solvent with the external protruding end fitted with a similar adaptor allowing connection to a vacuum line (Fig. 1E). Ground operculum samples were added to the glass pipette which was connected to the apparatus. Three mL of 25% ethanol was added to the vial to trap the volatile compounds before sealing. The opercula samples were volatilised by heating the body of the pipette containing the sample with the flame of a handheld butane torch. Vacuum pressure (700 mbar) was applied to the vial headspace via the steel tube above the solvent to bubble the smoke produced in the pipette through the trapping solvent.

Prior to burning each opercula sample, a blank sample control was collected to check for any background compounds present inside the pipette. For the blank sample, an empty glass pipette was connected and heated with the butane torch (Fig. 1E) from the outer surface for 1 min and the blank solvent was stored in an auto sampler vial for further analysis by GC-MS. No volatile or other bioactive compounds were observed in the blank solvent samples used in the study. For opercula smoke samples, 0.1 g of powdered opercula was added into the same glass pipette used for the blank sample and then burned by applying heat to the outer surface with the butane torch for 1 min. Smoke released from the opercula was bubbled through ethanol (25%). The ethanol solvent containing smoke water was stored in auto sampler GC vials for further analysis by GC-MS. For the *D*. *orbita* samples, this smoke collection process was repeated for three replicate samples (n = 3) of untreated opercula and opercula treated with ethanol and acetic acid, with their respective background sample.

### Preparation of lipophilic and polar operculum extracts

For preparing sequential lipophilic (hexane) and polar (methanol) solvent extracts (Fig. 1E), 10 grams of *D*. *orbita* opercula were used. Opercula were soaked in 100 ml of hexane for 24 hours then the solvent was decanted and replaced with new solvent until no further visible colour was extracted. The hexane extract was transferred to round bottom quick fit flasks and dried using a rotary evaporator (Buchi R114,) under vacuum pressure. Reduced extracts were transferred to amber vials (Agilent) using minimal volumes of hexane and dried under a stream of high purity nitrogen gas until no solvent remained. After hexane extraction, the opercula were subsequently soaked with 100 ml of methanol for 24 hours, then decanted and replaced with new methanol solvent until no further colour was extracted from the opercula. The methanol extracts were also dried using a rotary evaporator (Buchi R114; Chadderton, UK) and the reduced extract was transferred to amber vials and dried under a stream of N_2_. A small amount of N_2_ was added to the vials containing dried hexane and methanol extracts to prevent oxidation and the cap was fitted tightly and then wrapped with aluminium foil and stored in −20 °C until further analysis with LC-MS and GC-MS.

### Secondary metabolite analysis

*D*. *orbita* opercula dried extracts from hexane (0.213 g) and methanol (0.3204 g) were redissolved with 2 ml methanol. These extracts, as well as the smoke extracts trapped in ethanol (25%) from burning the ethanol, acetic acid treated and untreated opercula, were analysed using GC-MS, along with the respective blank samples. GC-MS was undertaken on Hewlett Packard (HP; Palo Alto, USA) 6890 GC coupled to an HP 5973 mass selective detector (MSD). The GC utilized a column of HP-5MS (Crosslinked 5% PH ME Siloxane) 30 m × 0.25 mm × 0.25 μm film thickness. All samples were analysed using a short run whereby the column temperature was controlled by HP ChemStation programmed from 50 °C (5 min held) to 250 °C (5 min held) @ 10 °C/min. The hexane extracts were analysed using an additional long run in which temperature gradient started at 50 °C was held for 5 min, then increased 4 °C/min up to 300 °C. In all cases the injection volume was 0.2 μL using an Agilent 7683 series injector with split ratio 1:10 and the injection port was set at 250 °C. Helium was used as the carrier gas with the flow rate of 0.7 ml/min. The source temperature in the MS detector was 250 °C, with an ion source voltage of 69.9 eV. The quadrupole was set at 150 °C and the transfer line held at 280 °C. Data processing was carried out using HP ChemStation software. Identification of volatile compounds was based on matches to the library mass spectra and fragmentation patterns (NIST02, WILEY 6, and ESSOILS mass spectral libraries; Gaithersburg, USA).

GC-MS analysis was also attempted for the *Chicoreus ramosus* smoke extracts but the samples were too dilute. Therefore a method was optimised using LC-MS for comparison of the smoke extracts from both *D*. *orbita* and *C*. *ramosus*. LC-MS analysis of the extracts was undertaken using an Agilent 1260 infinity high performance liquid chromatography (HPLC) system coupled to a 6120 Quad mass spectrometer. The HPLC utilized a Phenomenex luna C18 reversed phase column (100 × 4.6 mm with 2.6 μm 100 A; Torrance, USA) with a solvent gradient of 10 to 95% acetonitrile (ACN) with 0.005% trifluoroacetic acid (TFA), over 18 minutes at a flow rate of 0.5 mL/min. Peak absorption was monitored using serial UV/Vis diode-array detection (DAD). Electrospray ionisation (ESI) mass spectrometry was used in separate injections for positive and negative ion modes. The source temperature was 250 °C with potential applied at 3000 V. Selective ion monitoring (SIM) was undertaken at m/z 94 (phenol) and 109 (paracresol) in positive mode and at m/z 107 in negative mode. Mass scanning was undertaken from 90–1500 *m/z*. The opercula smoke samples were compared with reference standards of phenol (Sigma-Aldrich; 99% purity) and para cresol (Sigma-Aldrich; 99% purity) base on retention time, UV profile and SIM. Agilent ChemStation was used to analyse the LC-MS data, with chromatograms overlaid for the reference standards and the equivalent opercula smoke preparations from *D*. *orbita* and *C*. *ramosus*.

LC-MS was also used to analyse the hexane and methanol extracts from *D*. *orbita* opercula using the same conditions with mass scanning undertaken at 100–1500 *m/z* in positive and negative ion modes. The specific brominated indoles were identified by comparison of the retention times, UV profiles, molecular weight and fragmentation patterns reported in previous studies on *D*. *orbita* hypobranchial gland extracts^45,56,57^. In addition to mass detection from the total ion current, selected ion monitoring was undertaken in negative ion mode at *m/z* 224 and 226 for ^79^Br^, 81^Br isotopes to detect bromoisatin fragment ions [C_8_H_3_BrNO_2_]^−^ that are typicallly generated from brominated indoles including, tyrindoxyl sulfate, tyrindoleninone and tyriverdin^56^.

Identification of tyrindoxyl sulfate and murexine were determined by comparison of the HPLC retention time, UV trace and mass spectrum to compounds purified from the hypobranchial gland of *D*. *orbita*. and confirmed by nuclear magnetic resonance ^1^H-NMR^56,58^. Further comparision of the unidentified choline ester was done using pure standards for urocanic acid (4-Imidazoleacrylic acid) (Sigma; 99% purity) and choline bitartrate (Sigma; 99% purity) based on retention time UV profiles and mass spectra. The detection of adenosine and tryptophan were confirmed by comparison of the HPLC retention time, UV trace and mass spectral fragmentation with the pure standards of L-tryptophan (Sigma; with ≥98% purity) and adenosine (Olchemim; 98% purity).

The presence of murexine and other choline esters in the operculum extracts were further investigated by thin layer chromatography, as these polar compounds are known to be produced by *D*. *orbita* ^30^, and a large range of other muricids including species used in the traditional preparations^61^. The methanol extracts were spotted onto aluminium-backed silica gel plates (Merck), and separated using an n-butanol–EtOH–acetic acid–water (8:2:1:3) solvent system. The plates were then dipped in Dragendorff’s Reagent (Fluka-44578; St. Louis, USA), to allow visualization of alkaloids and quaternary ammonium bases^61^. Development of yellow and purple pigmentation in UV-active spots indicated the presence of murexine, choline and tyrindoxyl sulfate respectively^45,61^.

### Trace element analysis

The trace element composition (Ag, Ar, Pb, Cd, Cr, Cu, Mg, Ni, Se, Zn, Hg, Fe, Al, B, Si, V, Co, Mo, Ba, Ca, Mg, K, Na, S, P) of the dried muricid opercula power (Fig. 1A) was undertaken to check the potential health risk associated with direct ingestion of opercula. In total, 1 g of the untreated powdered opercula was analysed by the Environmental Analysis Laboratory (EAL), Southern Cross University (Laboratory Accreditation Number 14960). The samples were prepared using the hot-block acid digestion procedure^73^ and analysed by inductively coupled plasma - mass spectrometry (ICP-MS) using a NexION 300 D series ICP spectrometer with an ESI SC-FAST Auto Sampler (Perkin Elmer, Waltham, U.S.A.).

## Electronic supplementary material

Supplementary Information

## References

[1] Exodus. *The Old Testament*, *The Holy Bible: revised authorised version*. Samual Bagster & Sons Ltd (1982).

[2] Day, A. E. Onycha. In: *The International Standard Bible Encyclopedia*, *Volume 4* (ed. (eds Geoffrey, W. B.). Wm. B. Eerdmans Publishing Co. (1986).

[3] Abrahams HJ (1979). Onycha, ingredient of the ancient jewish incense: An attempt at identification. Econ Bot.

[4] Baker J (1974). Tyrian purple: an ancient dye, a modern problem. Endeavour.

[5] Sterman, B., Taubes-Sterman, J. *The Rarest Blue*. Lyons Press (2012).

[6] Cardon, D. *et al*. Who could wear true purple in Roman Egypt? Technical and social considerations on some new identifications of purple from marine molluscs in archaeological textiles. In: *Purpurae Vestes III*, *Textiles y Tintes en la Ciudad antigua* (ed. (eds Alfaro, C., Brun, J. P., Borgard, P. H., Benoit, R. P.). Universitat de València - Centre Jean Bérard (2011).

[7] Karapanagiotis I, Mantzouris D, Cooksey C, Mubarak MS, Tsiamyrtzis P (2013). An improved HPLC method coupled to PCA for the identification of Tyrian purple in archaeological and historical samples. Microchem J.

[8] Mchugh J (2013). Blattes de Byzance in India: Mollusc Opercula and the History of Perfumery. J R Asiat Soc.

[9] Easton, M. G. *Eastons Bible Dictionary from Illustrated Bible Dictionary*. *3rd Edition*. Thomas Nelson (1897).

[10] McClintock, J., Strong, J. *Cyclopaedia of Biblical*, *Theological*, *and Ecclesiastical Literature*. Harper & Brothers (1880).

[11] Cooksey, C. Tyrian purple: the first four thousand years. *Sci Prog* **96**, (2013). 10.3184/003685013X13680345111425 PMC10365538 23901634

[12] Sotiropoulou, S., Karapanagiotis, I. Conchylian purple investigation in prehistoric wall paintings of the Aegean area. In: *Indirubin*, *the red shade of Indigo* (ed. (eds Meijer, L, Guyard, N., Skaltsounis, L., Eisenbrand, G.). Life in Progress Editions (2006).

[13] Reese DS (1987). Palaikastro Shells and Bronze Age Purple-Dye Production in the Mediterranean Basin. The Annual of the British School at Athens.

[14] Reese, D. S. Iron Age shell purple-dye production in the Aegean. In: *Kommos IV: The Greek Sanctuary*, *Part 1*. (ed. (eds Shaw, J. W., Shaw, M. C.). Princeton University Press (2000).

[15] McGovern PE, Michel RH (1990). Royal purple dye: the chemical reconstruction of the ancient Mediterranean industry. Acc Chem Res.

[16] Allan J (1934). Tyrian purple. An ancient industry. Austral Mus Mag.

[17] Pulak, C. The Uluburun shipwreck and Late Bronze Age trade. In: *Beyond Babylon: Art*, *Trade*, *and the Diplomacy in the 2nd Millennium BC*. (ed. (eds Joan, A., Kim, B., Jean, M. E.). The Metropolitan Museum of Art (2008).

[18] Meyerhof, M., Sobhy, G. P. *The Abridged Version of “The book of Simple Drugs”*, *of Ahmad Ibn Muhammad Al-Ghafiqi by Gregorius Abul-Farag*. Al Ettemad Printing Press and Publising House (1932).

[19] Evershed RP, Van Bergen PF, Peakman TM, Leigh-Firbank EC (1997). Archaeological frankincense. Nature.

[20] Levey M (1961). Ibn Mäsawaih and his treatise on simple aromatic substances: Studies in the history of arabic pharmacology I. J Hist Med Allied Sci.

[21] Lipton, A. P., Syda, R. G., Jagadis, I. *The Indian Sacred Chank*. Central Marine Fisheries Research Institue (2013).

[22] Nawata, H. An exported item from Badi on the western Red Sea Coast in the eighth century: Historical and ethnographical studies on operculum as incense and perfume. In: *Ethiopia in Broader Perspective: Papers of 13th International Conference of Ethiopian Studies*. (ed. (eds Fukui, K., Kurimo, E.). Shokado Book Sellers (1997).

[23] Edward, J. K. P., Ayyakkannu, K. Shell trade and marketing with special reference to *Chicoreus ramosus* along the southeast coast of India. *Phuket Mar Biol Cent Spec Publ*, 33–34 (1992).

[24] Voultsiadou E (2010). Therapeutic properties and uses of marine invertebrates in the ancient Greek world and early Byzantium. J Ethnopharmacol.

[25] Lev E (2007). Drugs held and sold by pharmacists of the Jewish community of medieval (11–14th centuries) Cairo according to lists of *materia medica* found at the Taylor–Schechter Genizah collection, Cambridge. J Ethnopharmacol.

[26] Ahmad TB, Liu L, Kotiw M, Benkendorff K (2018). Review of anti-inflammatory, immune-modulatory and wound healing properties of molluscs. J Ethnopharmacol.

[27] Anand TP, Edward JP (2001). Screening for antibacterial activity in the opercula of gastropods. Phuket Mar Biol Cent Spec Publ.

[28] Murugan A, Ayyakannu K (1997). Operculum of *Chicoreus ramosus* and *Pleuroploca trapezium* a possible sources of bioactive substances. Phuket Mar Biol Cent Spec Publ.

[29] Benkendorff K (2015). Are the traditional medical uses of muricidae molluscs substantiated by their pharmacological properties and bioactive compounds?. Mar Drugs.

[30] Benkendorff K (2013). Natural product research in the Australian marine invertebrate *Dicathais orbita*. Mar Drugs.

[31] Benkendorff K (2010). Molluscan biological and chemical diversity: secondary metabolites and medicinal resources produced by marine molluscs. Biological Reviews.

[32] Tang R, Webster FX, Muller-Schwarze D (1993). Phenolic compounds from male castoreum of the Nother American beaver. Castor canadensis. J Chem Ecol.

[33] Opdyke, D. L. J. *Monographs on Fragrance Raw Materials: A Collection of Monographs Originally Appearing in Food and Cosmetics Toxicology*. Pergamon Press Ltd (1979).

[34] Dehn, D. L., Sullivan, J. B. J. Phenols and derivatives. In: *Clinical Environmental Health and Toxic Exposures 2nd edition* (ed. (eds Sullivan, J. B., Krieger, G. R.). Lippincott Williams & Wilkins (2001).

[35] Marteau C, Nardello‐Rataj V, Favier D, Aubry JM (2013). Dual role of phenols as fragrances and antioxidants: mechanism, kinetics and drastic solvent effect. Flavour Frag J.

[36] Butler A, Walker J (1993). Marine haloperoxidases. Chem Rev.

[37] NCBI. BioActivity Data for Compound 2-Chloro-4-methylphenol (CID 14851). (ed. (eds) National Center for Biotechnology Information, U.S. National Library of Medicine (2015).

[38] Mirghani, I, Nageeb, I. Local innovations and indigenous practices from the Sudan the hidden power of the poor. In: *Promoting Local Innovation in Sudan* (ed. (eds Mirghani I, Nageeb I). Prolinnova (2007).

[39] Carlson GP (1996). Comparison of the effects of pyridine and its metabolites on rat liver and kidney. Toxicol Lett.

[40] National Toxicology Program. NTP toxicology and carcinogenesis studies of pyridine (CAS No. 110-86-1) in F344/N rats, wistar rats, and B6C3F1 mice (drinking water studies). *National Toxicology Program Technical Report Series*, **470**, 1–330 (2000). 12579203

[41] WHO. *International Agency for Research on Cancer monographs on the evaluation of carcinogenic risks to humans*. World Health Organisation, International Agency for Research on Cancer (IARC) (2000).

[42] PansPantry. PansPantry multifaith incense trader “Onycha”. http://www.panspantry.co.uk/onycha.html Access Date: 20/06/2014 (2014).

[43] He G-Y (2013). Unexpected [M − H]+ ions in cyclopenta[*b*]indoles detection by electrospray ionization mass spectrometry. Journal of Mass Spectrometry.

[44] Benkendorff K, Bremner JB, Davis AR (2000). Tyrian purple precursors in the egg masses of the Australian muricid, *Dicathais orbita:* A possible defensive role. Journal of Chemical Ecology.

[45] Rudd D, Benkendorff K (2014). Supercritical CO_2_ extraction of bioactive Tyrian purple precursors from the hypobranchial gland of a marine gastropod. J Supercrit Fluids.

[46] Rudd, D. *et al*. Mass spectrometry imaging reveals new biological roles for choline esters and Tyrian purple precursors in muricid molluscs. *Scientific Reports* **5** (2015). 10.1038/srep13408 PMC4555103 26324173

[47] Cooksey CJ (2001). Tyrian purple: 6, 6′-dibromoindigo and related compounds. Molecules.

[48] Javier Lopez Chavez F, Rios Chavez P, Oyama K (2009). Brominated precursors of Tyrian purple (C.I. Natural Violet 1) from *Plicopurpura pansa*, *Plicopurpura columellaris* and *Plicopurpura patula*. Dyes and Pigments.

[49] Edwards V, Benkendorff K, Young F (2014). An *in vitro* high‐throughput assay for screening reproductive and toxic effects of anticancer compounds. Biotechnol Appl Biochem.

[50] Guan, H. S., Wang, S. G., H Yang, W J Z, Dong, W Yao In: Zhong Hua Hai Ben Cao [Chinese Marine MateriaMedica]. Shanghai Science and Technology Publishing House (2009).

[51] Lee KH, Wu YS, Hall IH (1977). Antitumor agents. 25. Synthesis and antitumor activity of uracil and thymine alpha-methylene- gamma-lactones and related derivatives. J Med Chem.

[52] NCBI. BioActivity Data for Compound Thymine (CID 1135). (ed. (eds). National Center for Biotechnology Information, U.S. National Library of Medicine (2015).

[53] Garratt C, Linker N, Griffith M, Ward D, Camm AJ (1989). Comparison of adenosine and verapamil for termination of paroxysmal junctional tachycardia. Am J Cardiol.

[54] Ratsch C, Muller-Ebeling C. *The Encyclopedia of Aphrodisiacs: Psychoactive Substances for Use in Sexual Practices*. Inner Traditions/Bear & Co (2013).

[55] Kelleher, W. J., McClintock, W. J. Anesthetic Oral Compositions Patent US 4931473A (1990).

[56] Valles-Regino R, Mouatt P, Rudd D, Yee L, Benkendorff K (2016). Extraction and quantification of bioactive Tyrian Purple precursors: A Comparative and validation study from the hypobranchial gland of a muricid *Dicathais orbita*. Molecules.

[57] Westley C, Benkendorff K (2008). Sex-specific Tyrian purple genesis: precursor and pigment distribution in the reproductive system of the marine mollusc, *Dicathais orbita*. J Chem Ecol.

[58] Esmaeelian B, Abbott C, Le Leu R, Benkendorff K (2014). 6-Bromoisatin found in muricid mollusc extracts inhibits colon cancer cell proliferation and induces apoptosis, preventing early stage tumor formation in a colorectal cancer rodent model. Mar Drugs.

[59] Meijer L (2003). GSK-3-selective inhibitors derived from Tyrian purple indirubins. Chem Biol.

[60] Sato N, Meijer L, Skaltsounis L, Greengard P, Brivanlou AH (2004). Maintenance of pluripotency in human and mouse embryonic stem cells through activation of Wnt signaling by a pharmacological GSK-3-specific inhibitor. Nat Med.

[61] Roseghini M, Severini C, Erspamer GF, Vittorio E (1996). Choline esters and biogenic amines in the hypobranchial gland of 55 molluscan species of the neogastropod Muricoidea superfamily. Toxicon.

[62] Cronstein BN (1994). Adenosine, an endogenous anti-inflammatory agent. J Appl Physiol.

[63] Whittaker V (1959). Acrylylcholine: a new naturally occurring pharmacologically active choline ester from *Buccinum undatum*. Biochem Pharmacol.

[64] Ronci M, Rudd D, Guinan T, Benkendorff K, Voelcker NH (2012). Mass spectrometry imaging on porous silicon: investigating the distribution of bioactives in marine mollusc tissues. Anal Chem.

[65] Duke C, Eichholzer J, Macleod J (1981). The synthesis of the isomeric N-methyl derivatives of murexine. Aust J Chem.

[66] Zhong Hua Ben Cao. *China State Administration Traditional Chinese Materia Medica Editorial Board*. *Zhong Hua Ben Cao* (*The Chinese Materia Medica*). Shanghai Science and Technology Publishing House (1999).

[67] Blackmore G, Wang W-X (2004). The transfer of cadmium, mercury, methylmercury, and zinc in an intertidal rocky shore food chain. J Exp Mar Bio Ecol.

[68] Fabris G, Turoczy NJ, Stagnitti F (2006). Trace metal concentrations in edible tissue of snapper, flathead, lobster, and abalone from coastal waters of Victoria, Australia. Ecotoxicol Environ Saf.

[69] Sloth JJ, Larsen EH, Julshamn K (2005). Survey of inorganic arsenic in marine animals and marine certified reference materials by anion exchange high-performance liquid chromatography-inductively coupled plasma mass spectrometry. J Agric Food Chem.

[70] FSANZ. Contaminants and Natural Toxicants. In: *Australia New Zealand Food Standards Code - Standard 1*.*4*.*1* http://www.foodstandards.gov.au/code/Pages/default.aspx. 01/03/2016 edn (2016).

[71] Ghiretti F (1994). Bartolomeo Bizio and the rediscovery of Tyrian purple. Experientia.

[72] Fowler MD (1985). Excavated Incense Burners: a case for identifying a site as sacred?. Palest Explor Q.

[73] Rayment, G. E., Lyons, D. J. *Soil Chemical Methods- Australasia*. CSIRO Publishing (2011).

[74] Aftel, M. *Fragrant: The Secret Life of Scent*. Penguin (2014).

[75] Etsy. C N Essential oil, Seashells, Wild Harvest, India https://www.etsy.com/au/listing/183508672/choya-nakh-essential-oil-seashells-wild) (2015).

[76] WoRMS Editorial Board. World Register of Marine Species. http://www.marinespecies.org at VLIZ (2017).

[77] Anthony, V. M. *et al*. Pyridine derivatives and their uses as fungicides and insecticides. Patent US 4826531A (1989).

[78] Othmer, K. *Kirk-Othmer Encyclopedia of Chemical Technology*, *5th Edition*. Wiley (2006).

[79] Johnson W (1993). Final report on the safety assessment of acetamide MEA. J Am Coll Toxicol.

[80] Strube A, Buettner A, Czerny M (2012). Influence of chemical structure on absolute odour thresholds and odour characteristics of ortho‐and para‐halogenated phenols and cresols. Flavour Frag J.

[81] Science lab. Material Safety Data Sheet Phenol MSDS. http://www.sciencelab.com/msds.php?msdsId=9926463. 21/05/2013 edn (2013).

[82] Bostanci S, Ekmekci P, Gurgey E (2001). Chemical matricectomy with phenol for the treatment of ingrowing toenail: a review of the literature and follow-up of 172 treated patients. Acta Derm Venereol.

[83] Science lab. Material Safety Data Sheet Para-Cresol MSDS. http://www.sciencelab.com/msds.php?msdsId=9923572. 21/05/2013 edn (2013).

[84] Blair RM (2000). The estrogen receptor relative binding affinities of 188 natural and xenochemicals: structural diversity of ligands. Toxicol Sci.

[85] ATSDR. Toxguide for Aluminum Al, B, V. (ed. (eds). Agency for Toxic Substances and Disease Registry,U.S. Department of Health and Human Services Public Health Service (2011).

