# Supplementary material for: Volatile and bioactive compounds in opercula from Muricidae molluscs supports their use in ceremonial incense and traditional medicines
Source: Sci Rep. 2017 Dec 12;7:17404. doi: 10.1038/s41598-017-17551-3 (PMC5727037; doi:10.1038/s41598-017-17551-3)
Supplement: Supplementary file 1 — Supplementary Information [file 41598_2017_17551_MOESM1_ESM.pdf]

The following supplement accompanies the article

Volatile and bioactive compounds in opercula from Muricidae molluscs supports their use in ceremonial incense and traditional medicines

Bijayalakshmi Devi Nongmaithem, Peter Mouatt, Joshua Smith, David Rudd, Michael Russell, Caroline Sullivan and Kirsten Benkendorff*

*Corresponding author: email kirsten.benkeodnrff@scu.edu.au

Scientific Reports XXX (2017)

Supplementary information

Supplementary Table 1 provides an overview of all the different compounds detected in the various preparations from the opercula and the mode(s) of detection. In total 33 distinct compounds were tentatively identified. The main bioactive compounds of interest were all detected by more than one analytical method.

In our study *Dicathais orbita* is used as a model species for the Muricidae family because it has been subject to a large number of previous studies specifically investigating Shellfish purple (6,6' dibromoindigo) and related precursor compounds that are characteristic of the Muricidae family (see reviews[29, 28]). However, as an Australian species, *D. orbita* was not used in the Mediterranean region in biblical times, for purple dyes or onycha. Consequently, we have undertaken some supplementary sampling of *Chicoreus ramosus* (sic *Murex inflatus*) that was historically available on trade routes and is still being traded today for use in incense and medicines. We were only able to obtain a small sample of three *C. ramosus* opercula but were able to divide these into the same pre-treatments as used for *D. orbita* smoke analysis and compare the extracts using LC-MS. This analysis confirmed that the opercula smoke of *C. ramosus* has a similar profile to the *D. orbita* extracts, with the presence of phenol and para cresol confirmed by comparison of the retention times and UV profile to reference standards (Figure S1 A, B & C). A dichlorinated phenol was also detected in the smoke extracts at r.t. 3.021 min (Fig. S1 A) with representative UV profile (Fig. S1 D) and *m/z* $[M-H]^-$ at 161, 163 and 165 in the expected isotopic ratio for a dichlorinated compound..

As phenol and cresol do not produce a signal in the TIC, either in the reference standards (Fig S2A) or extracts (Fig S2B&C), we used selected ion monitoring (SIM) at *mz* 94 for phenol and 104 for cresol. SIM effectively detected the reference compounds (Fig S2A) and provided very small signals for the relevant phenol and cresol peaks in both the *D. orbita* (Fig S2B) and *C. ramosus* (Fig. S2C) smoke extracts. It should be noted that as a result of other compounds that ionise more easily in the extracts, we could not increase the concentration any further without potentially overloading the detector.

Supplementary Table 1. Detected compounds from muricid opercula including mode of detection and major ions. OS, opercula smoke; E, ethanol; AA, acetic acid; U, untreated; OHE, opercula hexane extract; OME, opercula methanol extract. A * symbol indicates metabolites that exist as salt complexes and † symbol indicates where a reference standard was used for identification.

			**Method of analysis**								
			**GC-MS**			**LC-MS**					**TLC**
**Compound**	**Species**	**Extract (treatment)**	**R.T.**	**Ions detected**	**m/z**	**R.T**	**UV λ max (nm)**	**Mode, +(positive), /- (negative), Selected ions (SI)**	**Ions detected**	**m/z**	**R*f***
Pyridine	*D. orbita*,	OS (E, AA, U), OME	3.29	$[M]^{+\bullet}$	79						
Acetamide	*D. orbita*	OS (E, AA, U)	4.38	$[M]^{+\bullet}$	59, 44						
Chloro-phenol	*D. orbita*	OS (E, AA, U)	9.40	$[M]^{+\bullet}$	130, 128, 100						
Phenol	*D. orbita*, C. *ramosus*	OS (E, AA, U)	9.45	$[M]^{+\bullet}$	94	5.48†	212,270	UV, SI	[M]+	94	
Cresol	*D. orbita*, *C. ramosus*	OS (E, AA, U)	11.23	$[M]^{+\bullet}$	108, 107, 90	7.17†	220, 278	UV, ESI + MS, SI	[M+H]+	109, 94	
Chloro-methylphenol	*D. orbita*	OS (E, AA, U)	11.52	$[M]^{+\bullet}$	144, 142, 107						
Dichloro-phenol	*D. orbita* *C. ramosus*	OS (E, AA, U)	13.30	$[M]^{+\bullet}$	166, 164, 162	3.02	202,332	UV, ESI - MS	$[M-H]^{-}$,	165, 163, 161	
Cholestadiene	*D. orbita*	OHE	67.99	$[M]^{+\bullet}$	368, 353, 147						
Cholesterol	*D. orbita*	OHE	72.36	$[M]^{+\bullet}$	386, 368, 301, 275						
Dimethoxy butane	*D. orbita*	OHE	3.4	$[M-CH_3]^{+}$	103, 89, 87						

Myristic acid (tetradecanoic acid)	*D. orbita*	OHE	20.27	$[M]^{+\bullet}$	228, 185						
Palmitic acid	*D. orbita*	OHE	21.18	$[M]^{+\bullet}$	256, 227						
Oleic acid	*D. orbita*	OHE	23.99	$[M-H_2O]^+$	264, 220						
Stearic	*D. orbita*	OHE	24.19	$[M]^{+\bullet}$	284, 241						
Tyrindoleninone	*D. orbita*	OHE	21.18	$[M]^{+\bullet}$	255, 257	16.119	248, 402	UV, ESI + MS, SI	$[M]^+$, $[M-H]^+$	255, 257 254, 256	-
Tyriverdin	*D. orbita*	OHE				19.665	252, 402	UV, ESI + MS	$[M+Na]^+$	534, 536, 538	
Tyrindoxyl sulfate*	*D. orbita*	OHE				15.114 †	228, 302	UV, ESI – MS, SI	$[M]^-$	336,338	0.23
Murexine*	*D. orbita*	OME				2.041, 4.860†	266	UV, ESI + MS	$[M]^+$	224, 165, 159	0.29
Choline*	*D. orbita*	OME				2.041†	No UV	UV, ESI + MS	$[M]^+$	104	0.32
Urocanic acid (murexine fragment)	*D. orbita*	OME				2.041†	266	UV, ESI + MS	$[M+H]^+$ ($[M]^+$)	139 (138)	-
Adenosine	*D. orbita*	OME				2.84†	204, 258	UV, ESI + MS	$[M+H]^+$	268, 136, 144	-
Trytophan	*D. orbita*	OME				3.321†	218, 278	UV, ESI + MS	$[M+H]^+$	205, 188	-
Propenoic acid	*D. orbita*	OME	2.86	$[M]^{+\bullet}$	72, 55, 45						
Methyl pyroglutamate	*D. orbita*	OME	15.81	$[M]^{+\bullet}$	143, 115, 84						
Bipyridine	*D. orbita*	OME	17.87	$[M]^{+\bullet}$	156						
Thymine	*D. orbita*	OME	18.67	$[M]^{+\bullet}$	126						
Adenine	*D. orbita*	OME	20.43	$[M]^{+\bullet}$	135, 119, 108						
Dimethyl hypoxanthine	*D. orbita*	OME	21.64	$[M]^{+\bullet}$	164, 149						

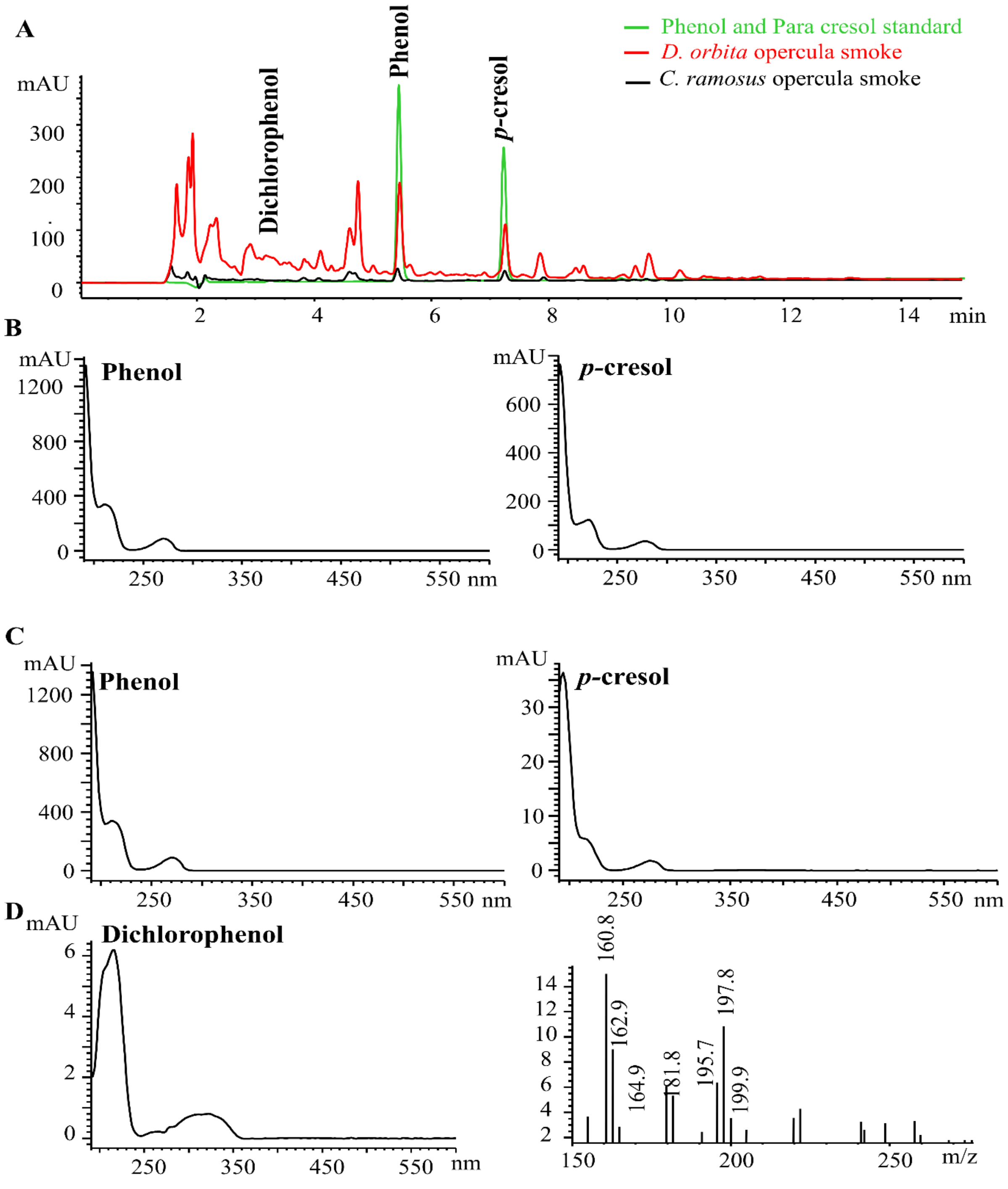

Figure S1 | Phenols in the opercula smoke samples from *Chicoreus ramosus* (black lines) and *Dicathais orbita* (red lines) in comparison to reference standards (green lines) using liquid chromatography mass spectrometry. A) HPLC chromatograms from the diode array detected at 210 nm. The X axis represents the retention time (minutes) in the C18 column before the compounds were detected. Y axis represents the absorbance units (mAU) from the diode array; B) UV-Vis profile of reference standards phenol and para cresol; C) UV-Vis profile of phenol (r.t 5.48 min) and para cresol (r.t 7.17 min) in a representative opercula smoke sample from *D. orbita;* and D) UV-Vis profile and mass spectrum of dichlorophenol (r.t. 3.021 min) in the smoke extracts.

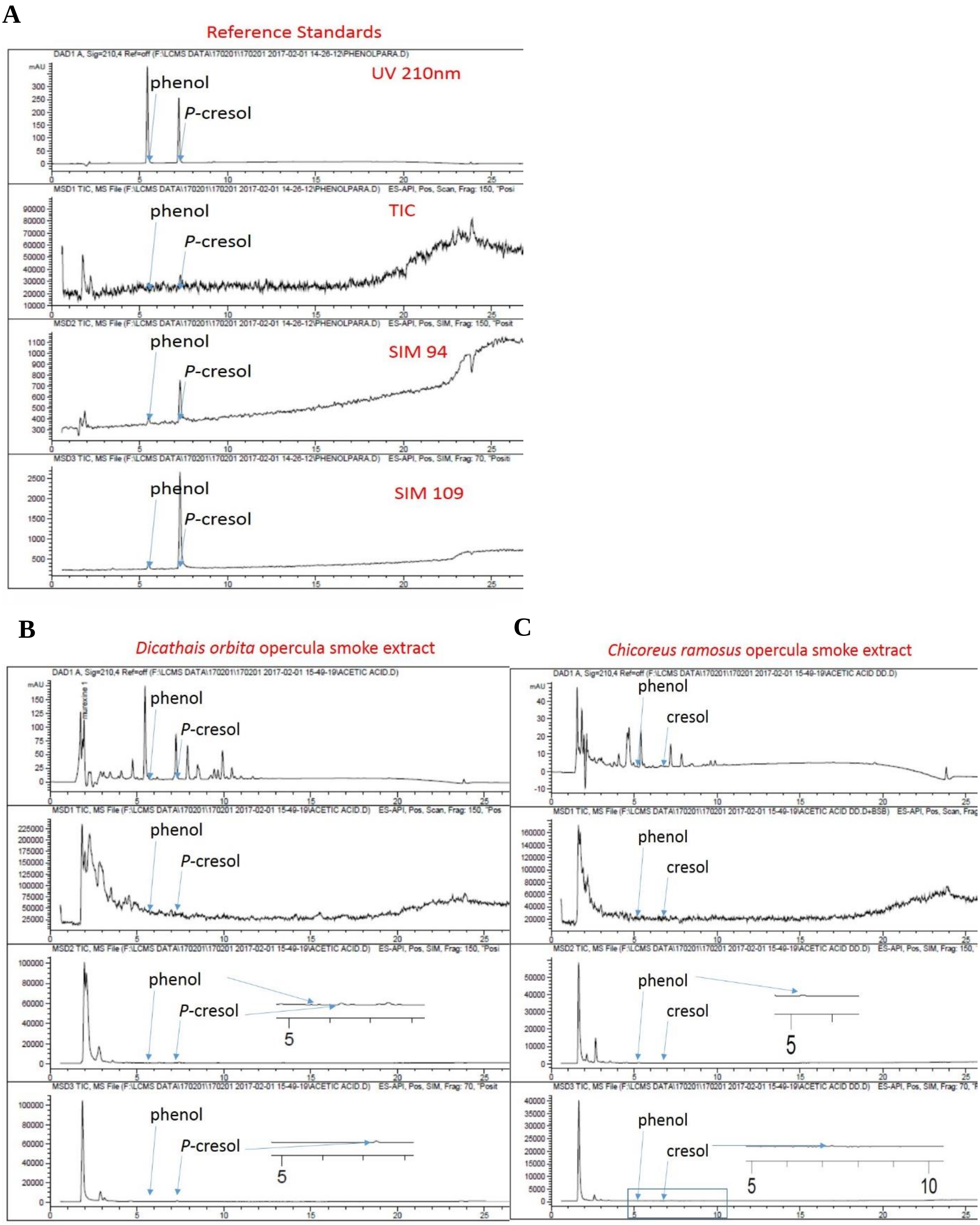

Figure S2 | LCMS detection of phenol and cresol showing chromatograms for UV at 210nm, total ion current (TIC) and selective ion monitoring at *m/z* 94 and 109; A) analytical reference standards; B) smoke extracts from *D. orbita* opercula and C) smoke extracts from *C. ramosus* opercula.

Further evidence for the presence of cholinated phenols in the smoke extracts form *D. orbita* opercula was provided by GC-MS analysis. The NIST library matches for chlorophenol, methylchlorophenol and a dichlorophenol against compounds detected in the smoke extracts are provided in Fig. S3.

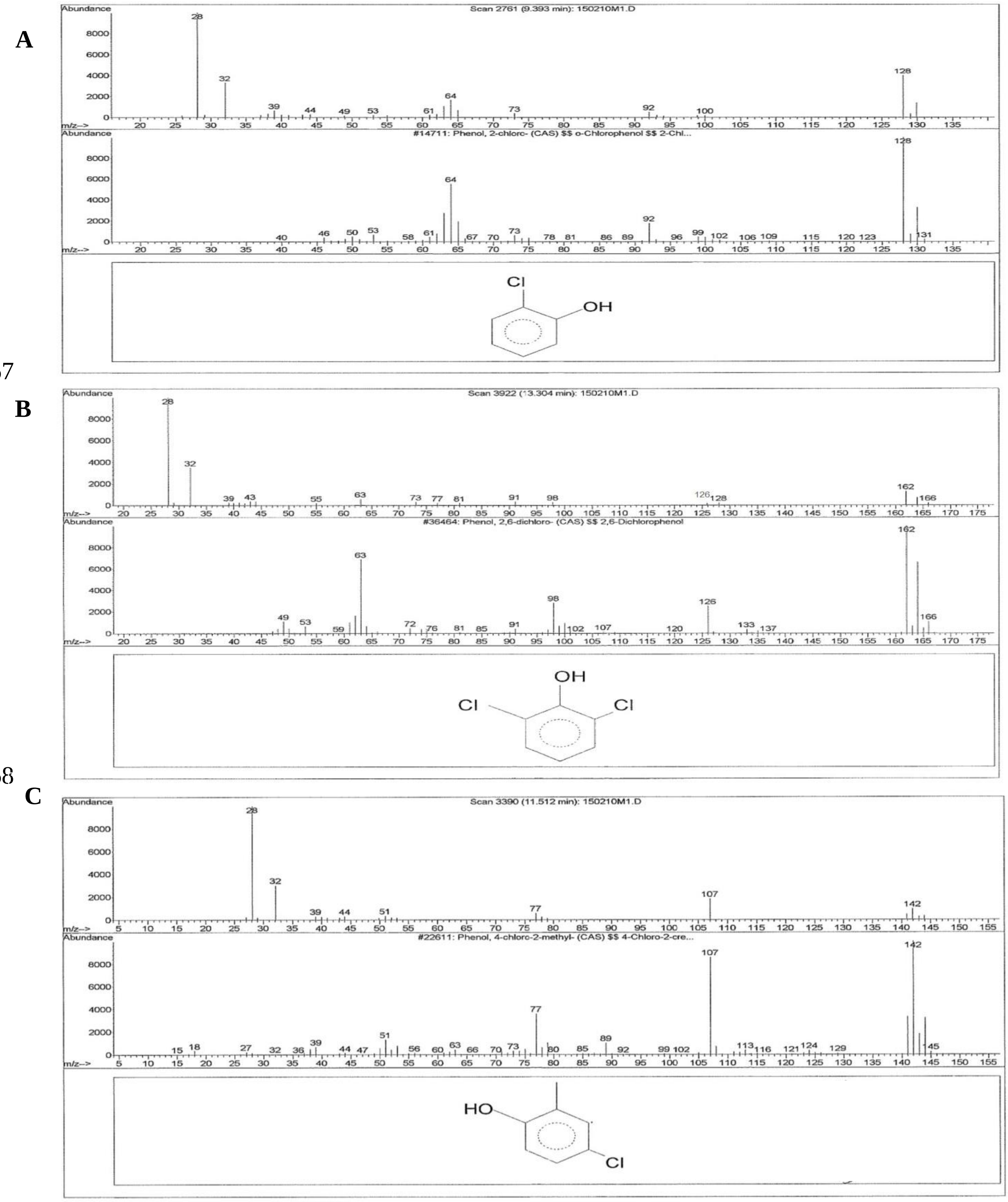

Figure S3: **Mass spectra of chlorinated phenols detected by GC-MS** in the smoke extracts from *D. orbita* opercula, showing matches to the NIST library for; A) chlorophenol; B) methylchlorophenol and C) dichlorophenol.

To examine the lipophylic compounds likely to be extracted in oil, we used hexane extracts of *D. orbita* operculum. GC-MS analysis of the hexane extracts revealed the presence of predominately saturated fatty acids, the monounsaturated omega-9 fatty acid oleic acid and small amounts of dimethoxy butane (Table S2). The brominated indole tyrindoleninone was detected as a minor component (Figure S4). To examine the polar based compounds, we used methanol extract of *D. orbita* operculum. The dominant compounds detected by GC-MS were propenoic acid and methyl pyroglutamate, but the methanol extract also shows the presence of pyridine, bipyridine and dimethyl hypoxanthine, as well as the nucleobases thymine and adenine (Table S2). The preliminary run of hexane extract was undertaken using a temperature gradient starting with oven temperature of 50 °C held 5 minutes, then increasing 4 °C/min up to 300 °C and with the total run time of 74.5 minutes. This method produced the same result as the short run described in the main paper method section, except for the detection of cholestenol, which was not detected in the short run. The short run also detect some extra fatty acids; oleic acid and myristic (Table S2). Here we have represented the data from the short run for consistency with the methanol extract results in the main paper.

Table S2 | GCMS results of the muricid *Dicathais orbita* opercula extract.

Compound	Retention time (min)	Major m/z	Area %	Bioactive properties
**Hexane extract**				
2,2 dimethoxy butane	3.4	103, 89, 87	0.23	antifungal [67, 68]
myristic acid	20.27	228, 185	1.40	-
tyrindoleninone	21.18	240, 257	0.22	antimicrobial[36], anticancer[69, 70] steroidogenic activity[21, 35]
palmitic acid	22.31	256, 227	11.51	common primary metabolite
oleic acid	23.99	264, 220	6.48	-
stearic acid	24.19	284, 241	5.32	common primary metabolite
**Methanol extract**				
propenoic acid	2.86	74,72, 69	15.74	no detrimental health effects[71]
pyridine	3.32	79, 75	10.08	Refer to Table 2
methyl pyroglutamate	15.81	143, 115, 84	12.44	-
bipyridine	17.87	159, 156	1.68	-
thymine	18.67	170, 126	2.31	essential nucleic acid and active in NIH screen against mammary adenocarcinomas[39]
adenine	20.43	135, 119, 108	1.82	essential nucleotide and immunomodulatory[72]
dimethyl hypoxanthine	21.64	164, 149	4.53	alters antioxidant enzyme activity[73]

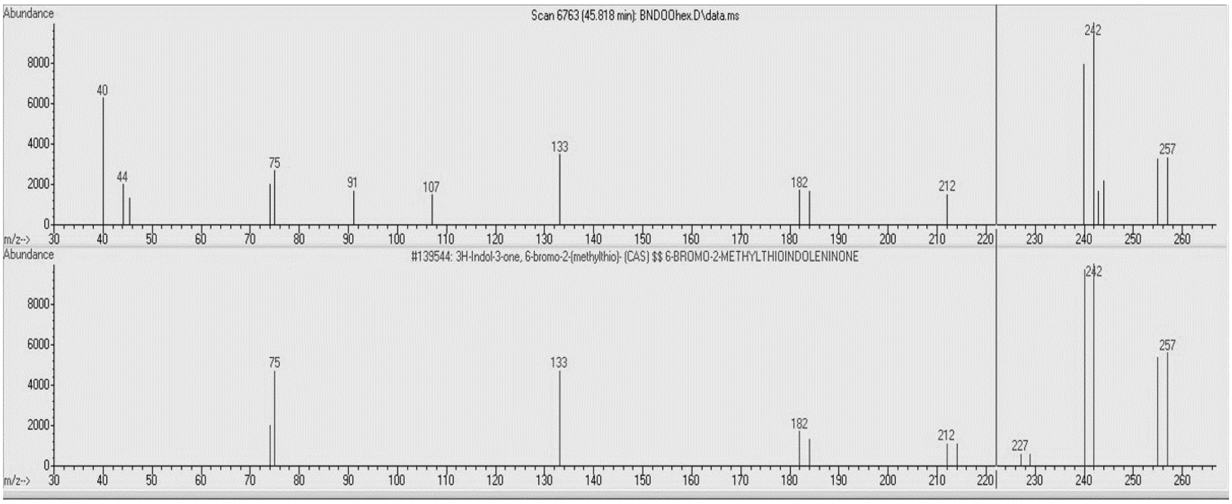

Figure S4: Mass spectra of tyrindoleninone detected by GC-MS in the hexane extract of *Dicathais orbita* operculum (top panel) showing the match to the WILEY 275 library (bottom panel).

LC-MS analyses of the hexane (Figure S5) and methanol (Figure S6) extracts from *D. orbita* opercula were undertaken using electron impact ionisation in in both positive and negative ion mode and selected ion monitoring at *mz* 224, 226 was used to detect potential brominated (Br^{79}, Br^{81}) indoles in negative ion mode. Tyrindoxyl sulfate, murexine and choline in the methanol extracts were also detected by thin layer chromatography with Dragendorff's reagent (Figure S7).

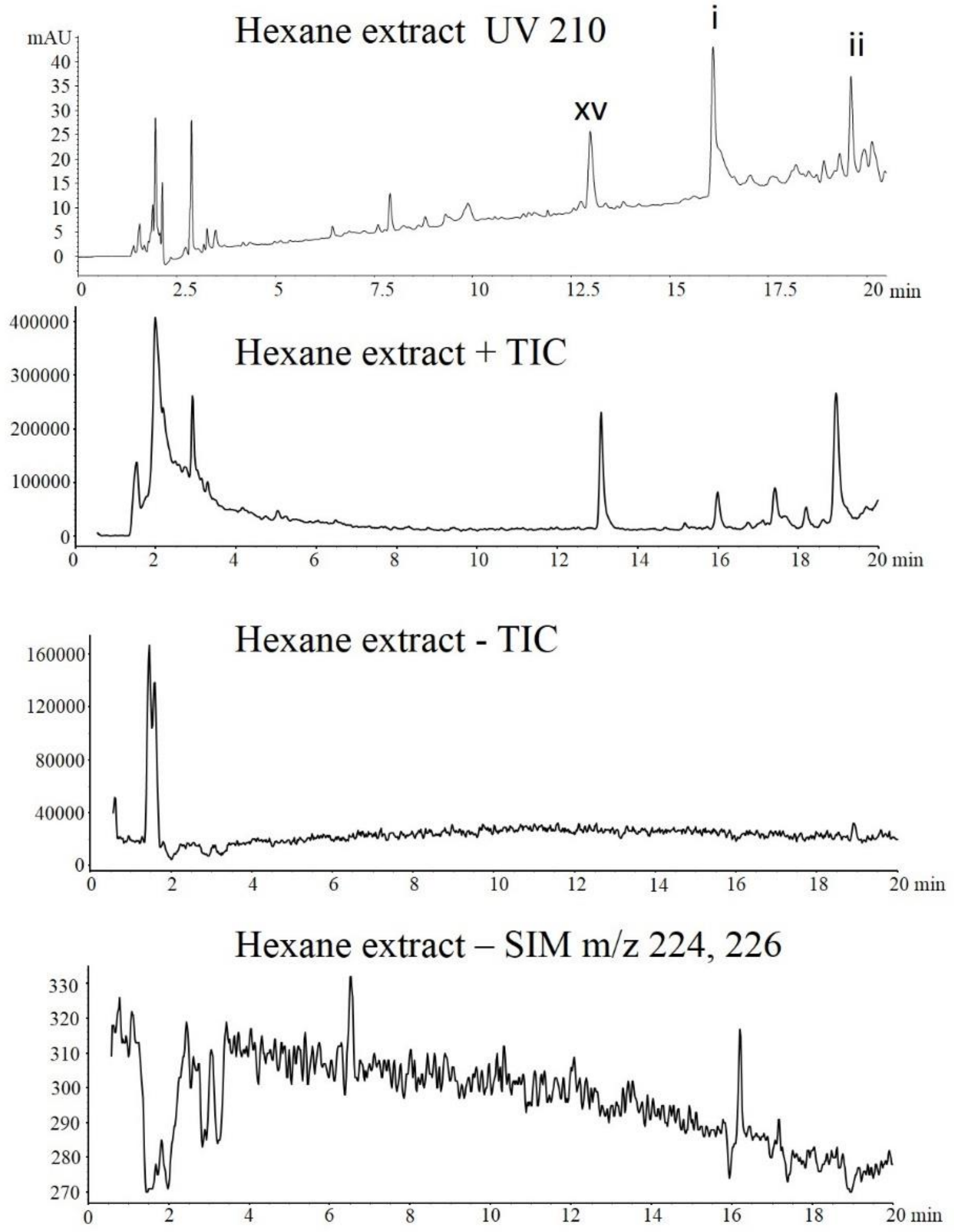

Figure S5: LC-MS of the hexane extract from *D. orbita* opercula, showing chromatograms for UV at 210nm, total ion current, in positive and negative mode and selective ion monitoring at *m/z* 224 and 226; i) tyrindoleninone; ii) tyriverdin; xv) unidentified compound.

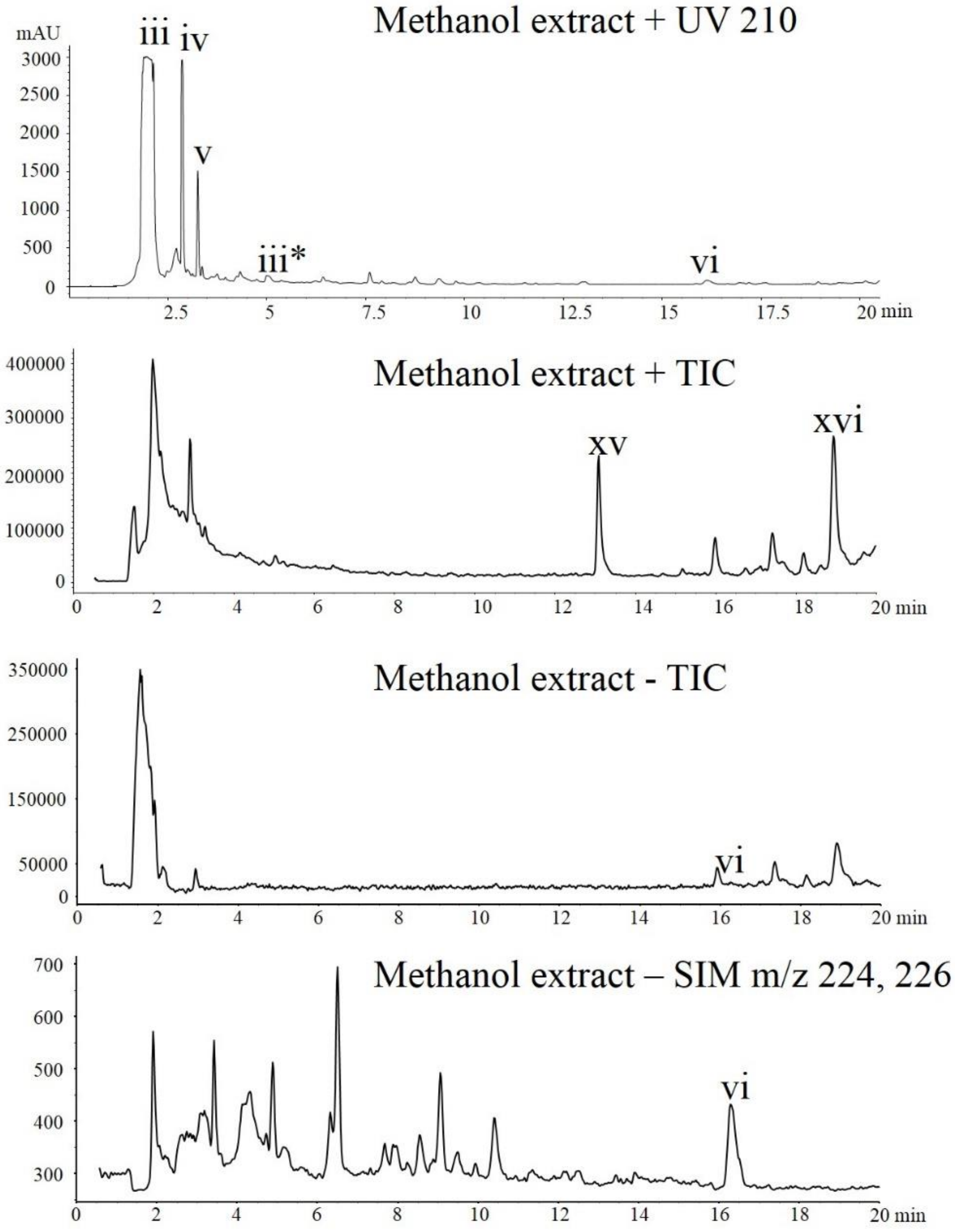

Figure S6: LC-MS of the methanol extract from *D. orbita* opercula, showing chromatograms for UV at 210nm, total ion current in positive and negative mode and selective ion monitoring at *m/z* 224 and 226; iii) murexine; iv) adenosine; v) tryptophan; xv & xvi) unidentified compounds.

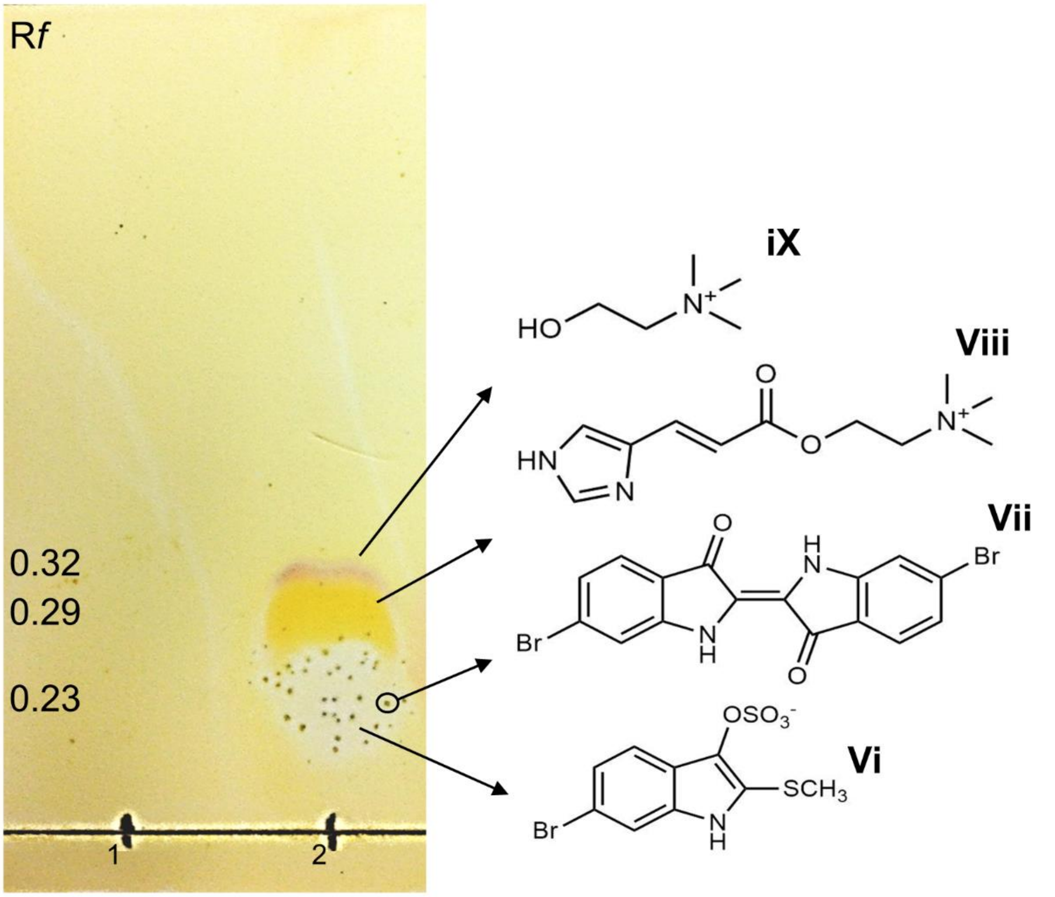

Figure S7 | Thin layer chromatography of alkaloids and quaternary ammonium bases on aluminium-backed silica plates visualized using Dragendorff reagent. Mobile phase was butanol-ethanol-acetic acid-water (8:2:1:3). Lanes include (1) control using acetone and (2) opercula methanol extract in acetone. R*f* 0.23 corresponds to tyrindoxyl sulfate (**vi**) where the spots turn to Shellfish purple (6,6'dibromoindigo **vii**) after exposure to HCl[51], R*f* 0.29 corresponds to murexine (**viii**) [56] and R*f* 0.32 corresponds to choline (**ix**)[58].

In addition to the compounds identified in Table S1, the LC-MS detected a number of compounds which have not yet been identified. The SIM 224, 226 chromatogram of the methanol extract detected a number of other compounds, which appear not to be brominated based on the mass ion ratios (Figure S8). The peak labelled xv detected around 13mins in positive TIC in both the hexane (Fig. S5) and methanol (Fig. S6) extracts produced an apparent molecular ion at *mz* 254 and appears to be highly unsaturated based on the UV profile (xv Figure S9). Another peak around 19 min in the positive TIC of the methanol extract, which appears to be a dimer *mz* 965 & 481.5 (xvi Fig. S9).

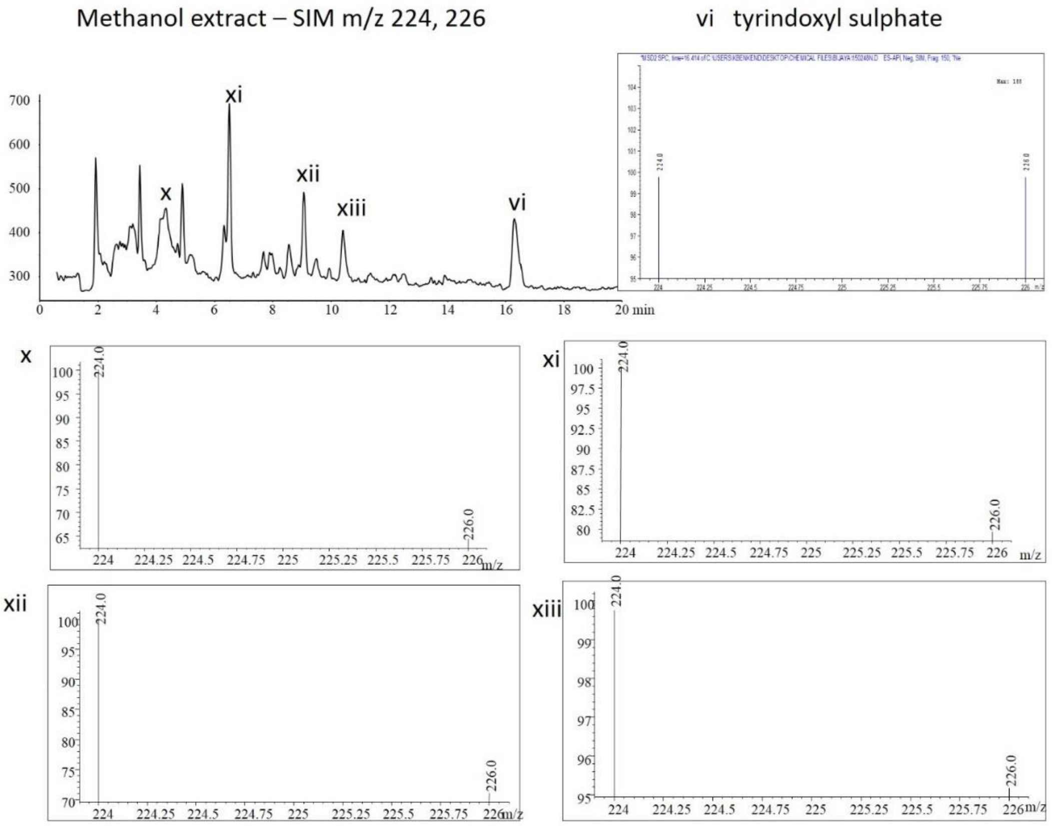

Figure S8: **Selected ion monitoring at 224 and 226 for potential brominated indoles in the methanol extract of *D. orbita* opercula**. Only tyridoxyl sulfate (vi) shows the expected 1:1 isotope ratio for Br^{79} Br^{81} . Representative spectra shown for peaks x, xi and xii are clearly not brominated. Peak xiii is potentially brominated but was only detected in trace amounts and did not produce a clear mass spectrum in the total ion current (Figure S5).

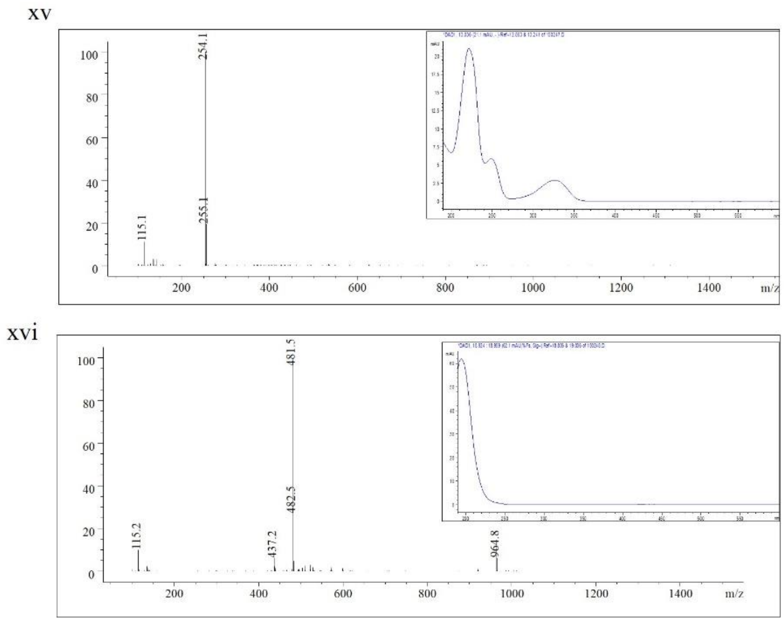

Figure S9: Mass spectrum and UV profile of unidentified peaks xv and xvi detected in the methanol extracts of *D. orbita* opercula (Figure S6).
